# Supplementary material for: Community health and human-animal contacts on the edges of Bwindi Impenetrable National Park, Uganda
Source: PLoS One. 2021 Nov 24;16(11):e0254467. doi: 10.1371/journal.pone.0254467 (PMC8612581; doi:10.1371/journal.pone.0254467)
Supplement: S1 Fig — A. The set up for washing hands and water usage outside a house. B. Water supply in plastic containers. C. Water supply from artesian well. D. Local butchers entrance. D. and E. goats very close to the park border. F. Cooking area in one of the village’s houses. Photos: D. Hayman. (DOCX) [file pone.0254467.s001.docx]

**Supporting Information**


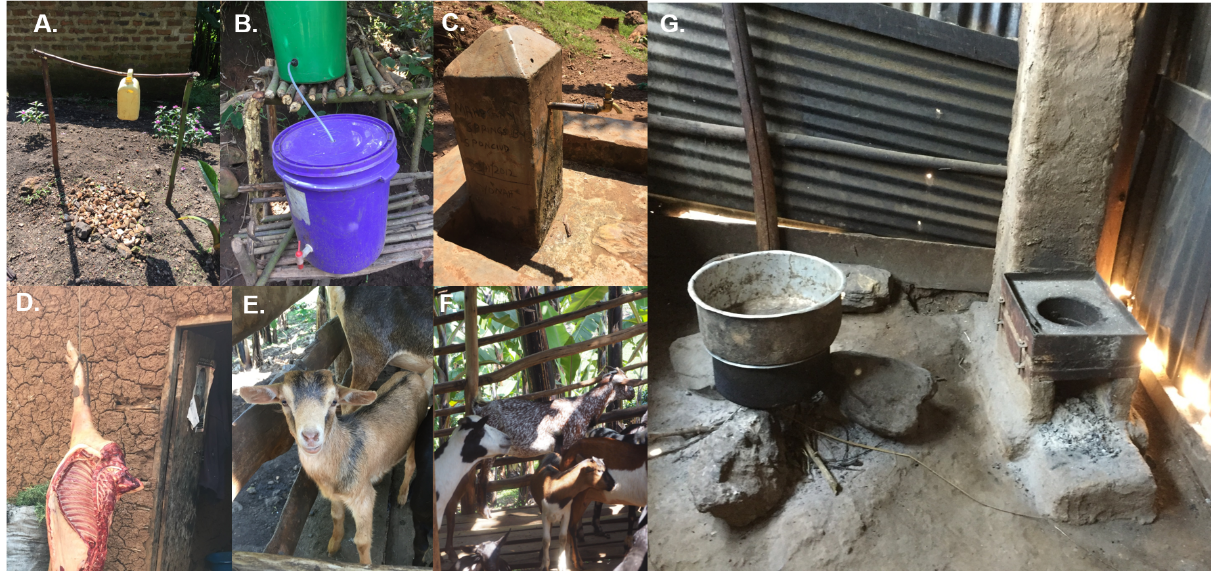


# **S1 Figure: Photos from Buhoma, Uganda, showing elements that are part of the local living conditions around Bwindi Impenetrable National Park.** A. The setup for washing hands and water usage outside a house. B. Water supply in plastic containers. C. Water supply from artesian well. D. Local butchers entrance. D. and E. goats very close to the park border. F. Cooking area in one of the village’s houses. Photos: D. Hayman.
