# Supplementary material for: Community health and human-animal contacts on the edges of Bwindi Impenetrable National Park, Uganda
Source: PLoS One. 2021 Nov 24;16(11):e0254467. doi: 10.1371/journal.pone.0254467 (PMC8612581; doi:10.1371/journal.pone.0254467)
Supplement: S2 Fig — A. Panoramic view of the street. B. View of the village close to the Bwindi Impenetrable National Park and livestock in the background. C. Closer view of one of the local houses. Photos: D. Hayman. (DOCX) [file pone.0254467.s002.docx]

**Supporting Information**


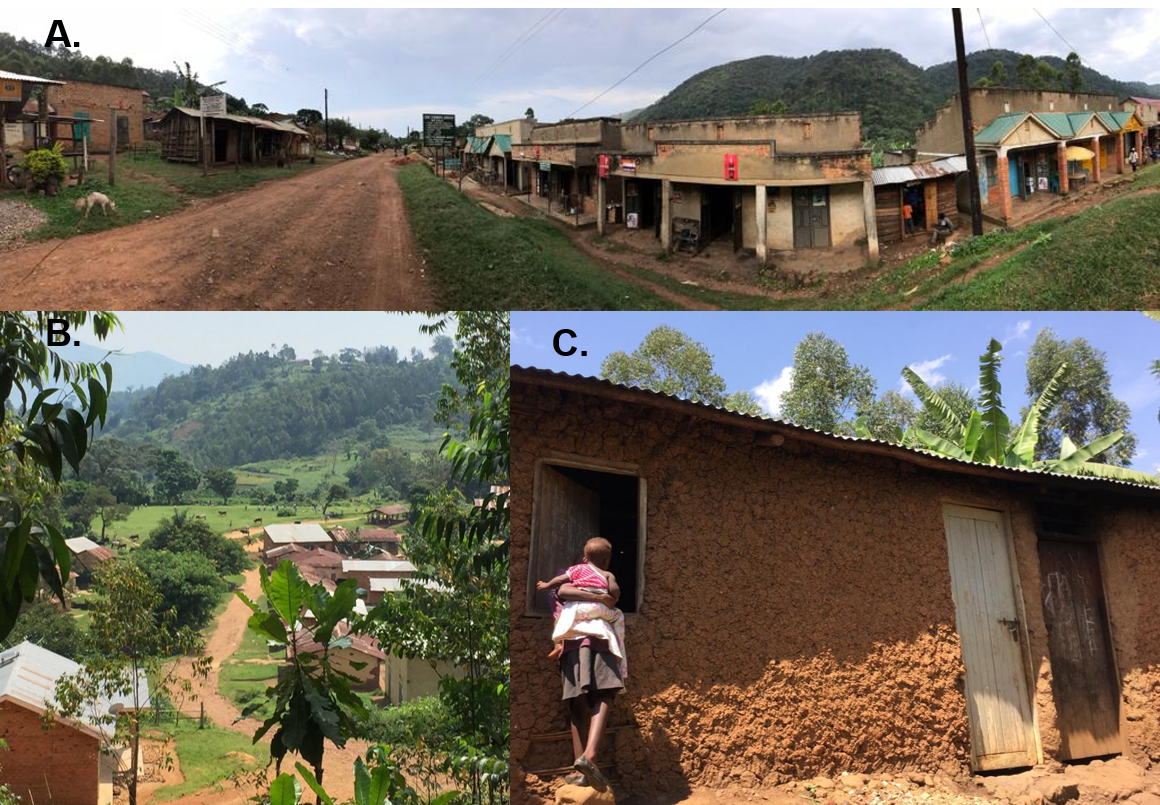


# **S2 Figure. Pictures from Buhoma, Uganda, showing parts of the village.** A. Panoramic view of the street. B. View of the village close to the Bwindi Impenetrable National Park and livestock in the background. C. Closer view of one of the local houses. Photos: D. Hayman.
