# Supplementary material for: Community health and human-animal contacts on the edges of Bwindi Impenetrable National Park, Uganda
Source: PLoS One. 2021 Nov 24;16(11):e0254467. doi: 10.1371/journal.pone.0254467 (PMC8612581; doi:10.1371/journal.pone.0254467)
Supplement: S3 Fig — Gender bias in our sampling is a significant study limitation. (DOCX) [file pone.0254467.s003.docx]

**Supporting Information**


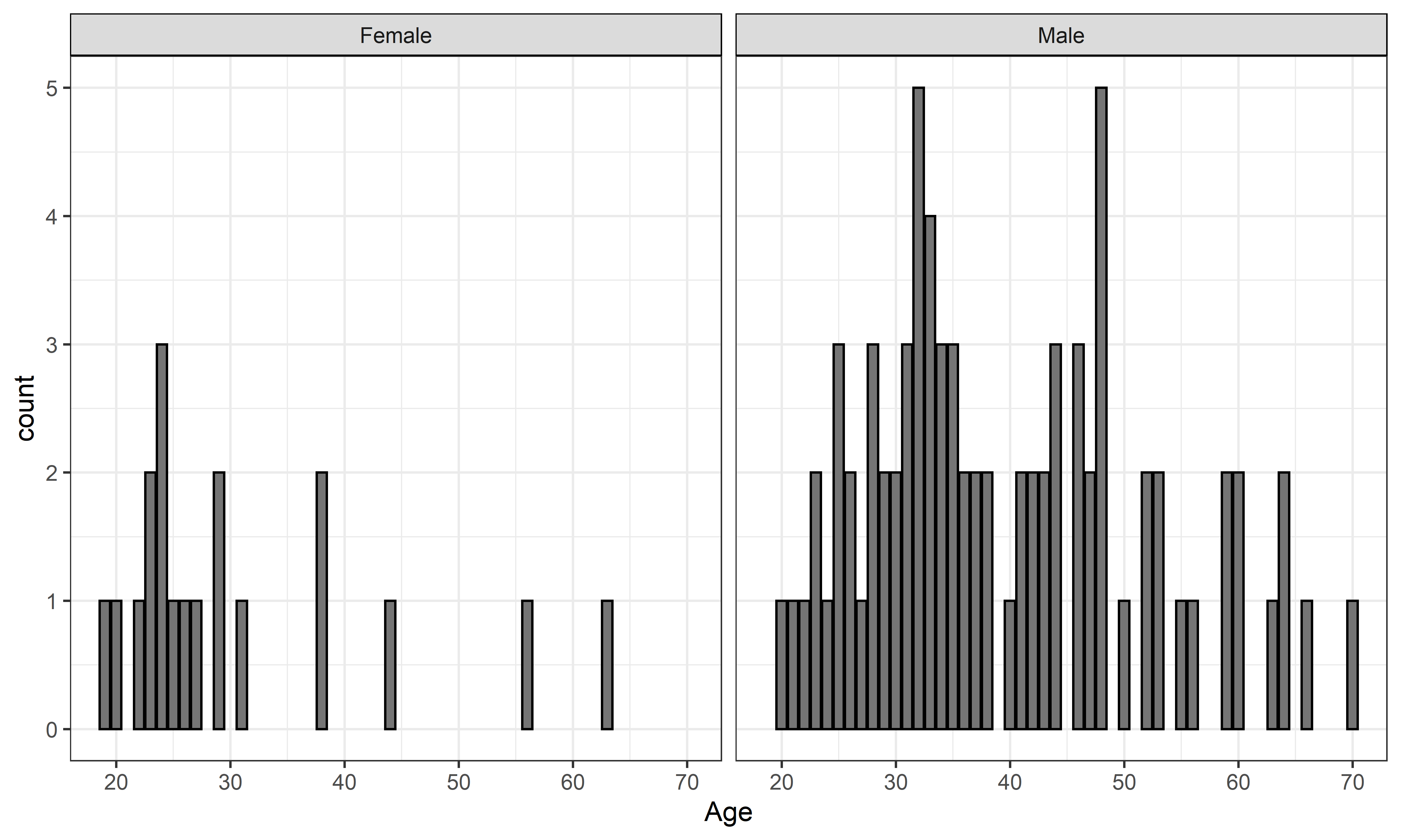


# **S3 Figure. Gender and age distribution of the participants.** Gender bias in our sampling is an important study limitation.
