# Supplementary material for: Community health and human-animal contacts on the edges of Bwindi Impenetrable National Park, Uganda
Source: PLoS One. 2021 Nov 24;16(11):e0254467. doi: 10.1371/journal.pone.0254467 (PMC8612581; doi:10.1371/journal.pone.0254467)
Supplement: S4 Fig — The average number of children per age group of participants varied from no children older than six years old for younger participants (between 16–20 years old) to more than three children for participants above 30 years old. (DOCX) [file pone.0254467.s004.docx]

**Supporting Information**


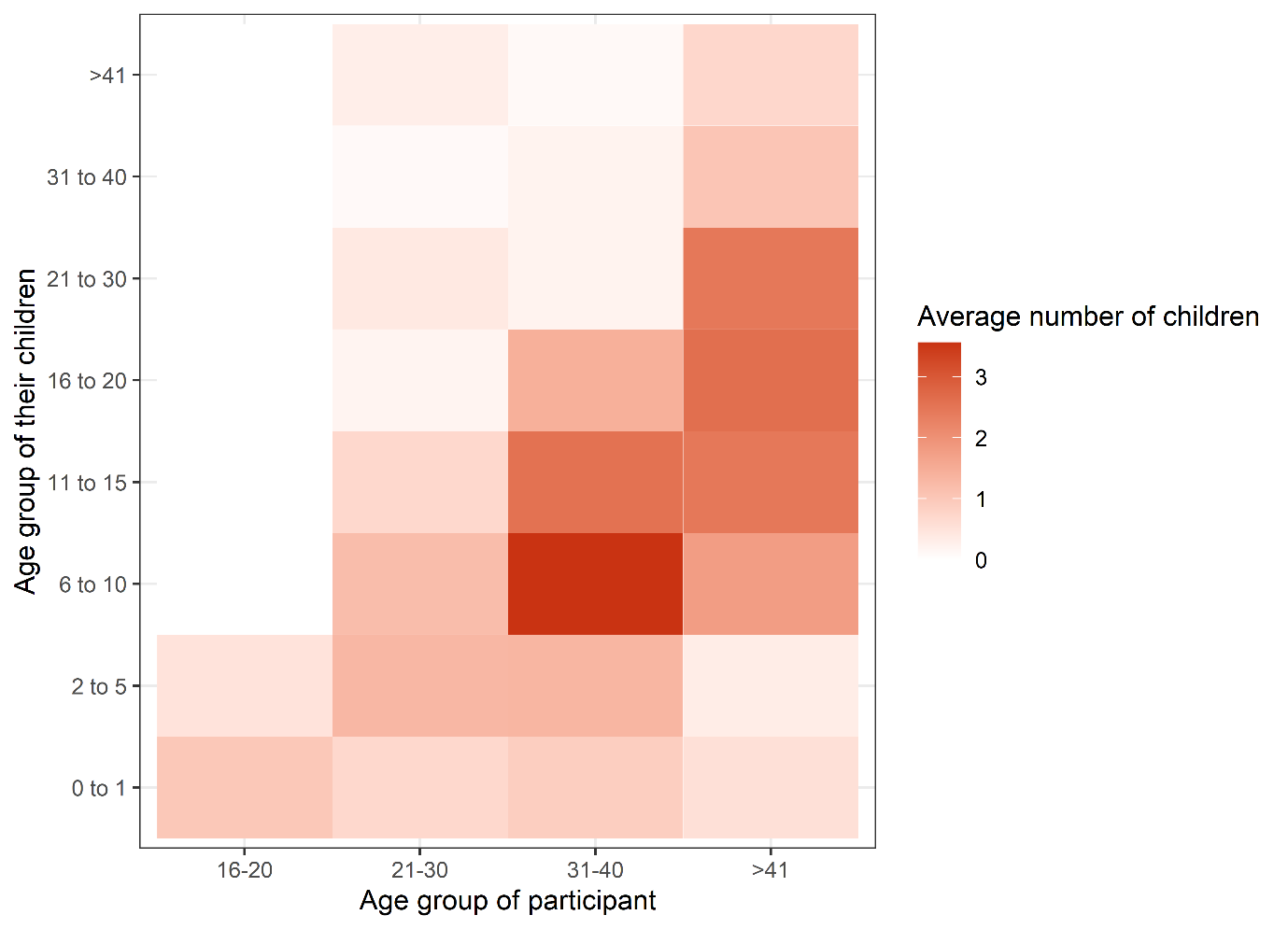


# **S4 Figure. Average number of children per participant age group.** The average number of children per age group of participants varied from no children older than six years old for younger participants (between 16-20 years old) to more than three children for participants above 30 years old.
