# Supplementary material for: Community health and human-animal contacts on the edges of Bwindi Impenetrable National Park, Uganda
Source: PLoS One. 2021 Nov 24;16(11):e0254467. doi: 10.1371/journal.pone.0254467 (PMC8612581; doi:10.1371/journal.pone.0254467)
Supplement: S5 Fig — Treatment choices of those who declared to be unwell or seek treatment (abbreviated as “treat.”) during the week before diary completion. (DOCX) [file pone.0254467.s005.docx]

**Supporting Information**


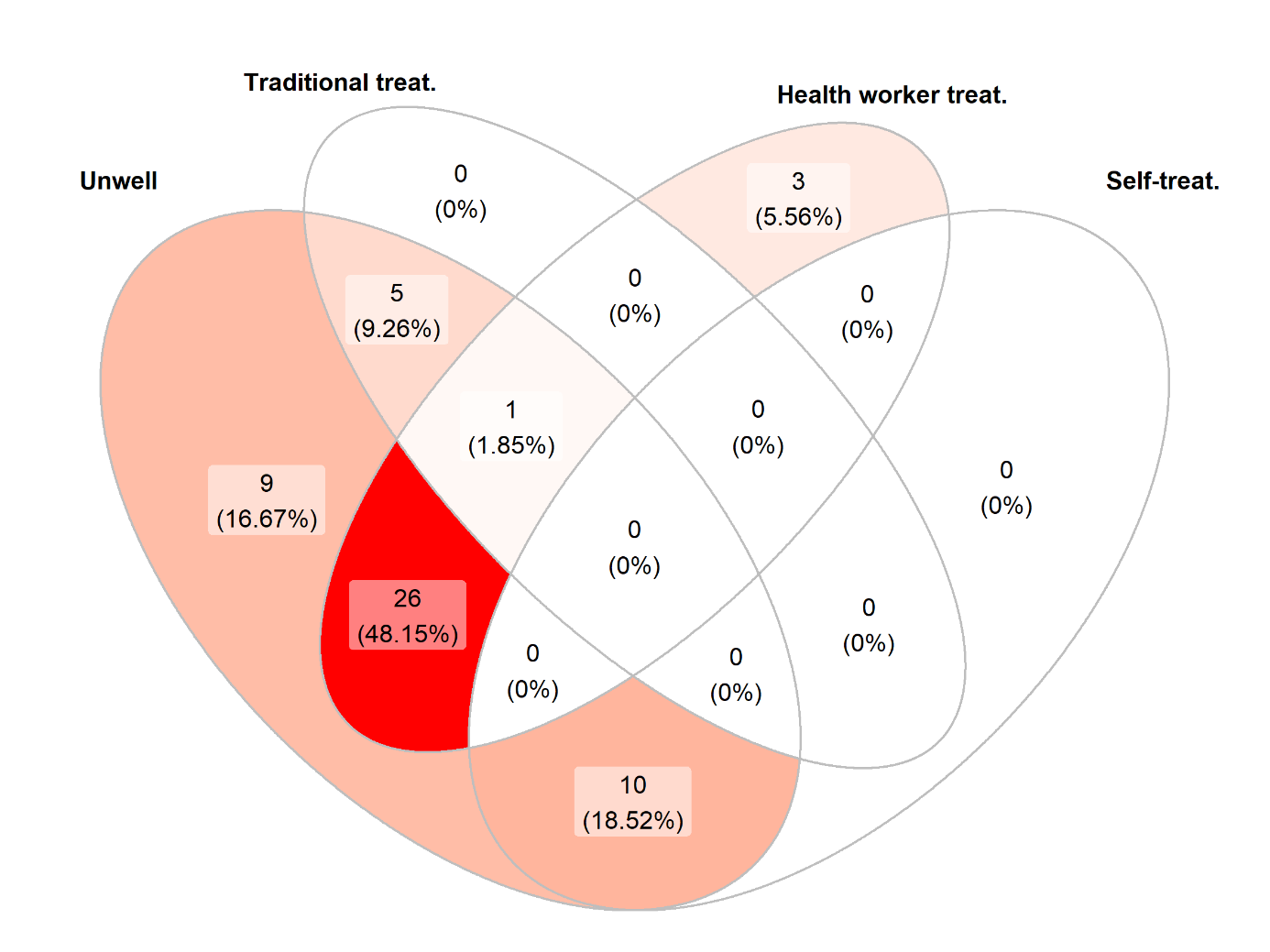


# **S5 Figure. Health care behaviours in Buhoma during a self-reported survey conducted in 2018.** Treatment choices of those who declared to be unwell or seek treatment (abbreviated as “treat.”) during the week before diary completion.
