# Supplementary material for: Community health and human-animal contacts on the edges of Bwindi Impenetrable National Park, Uganda
Source: PLoS One. 2021 Nov 24;16(11):e0254467. doi: 10.1371/journal.pone.0254467 (PMC8612581; doi:10.1371/journal.pone.0254467)
Supplement: S6 Fig — Observed contacts were collected throughout one week in a self-reported diary per age class. (DOCX) [file pone.0254467.s006.docx]

**Supporting Information**


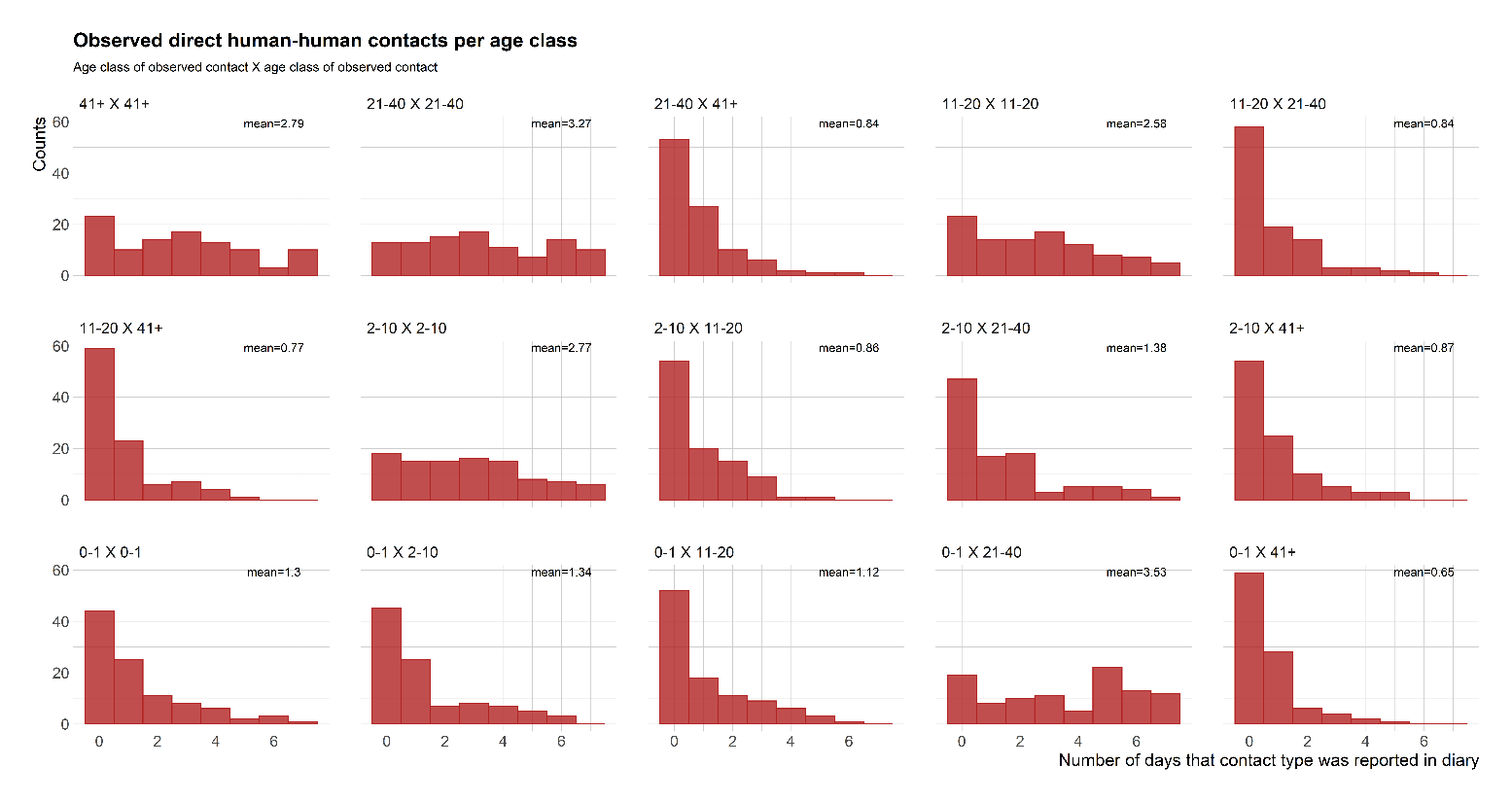


# **S6 Figure. Self-reported observed human-human contacts around BNP.** Observed contacts were reported throughout one week in a self-reported diary per age class.
