# Supplementary material for: Community health and human-animal contacts on the edges of Bwindi Impenetrable National Park, Uganda
Source: PLoS One. 2021 Nov 24;16(11):e0254467. doi: 10.1371/journal.pone.0254467 (PMC8612581; doi:10.1371/journal.pone.0254467)
Supplement: S7 Fig — Contacts were collected throughout one week in a self-reported diary. A. Sightings of dung in gray. B. Direct contact with dung in gray. (DOCX) [file pone.0254467.s007.docx]

**Supporting Information**


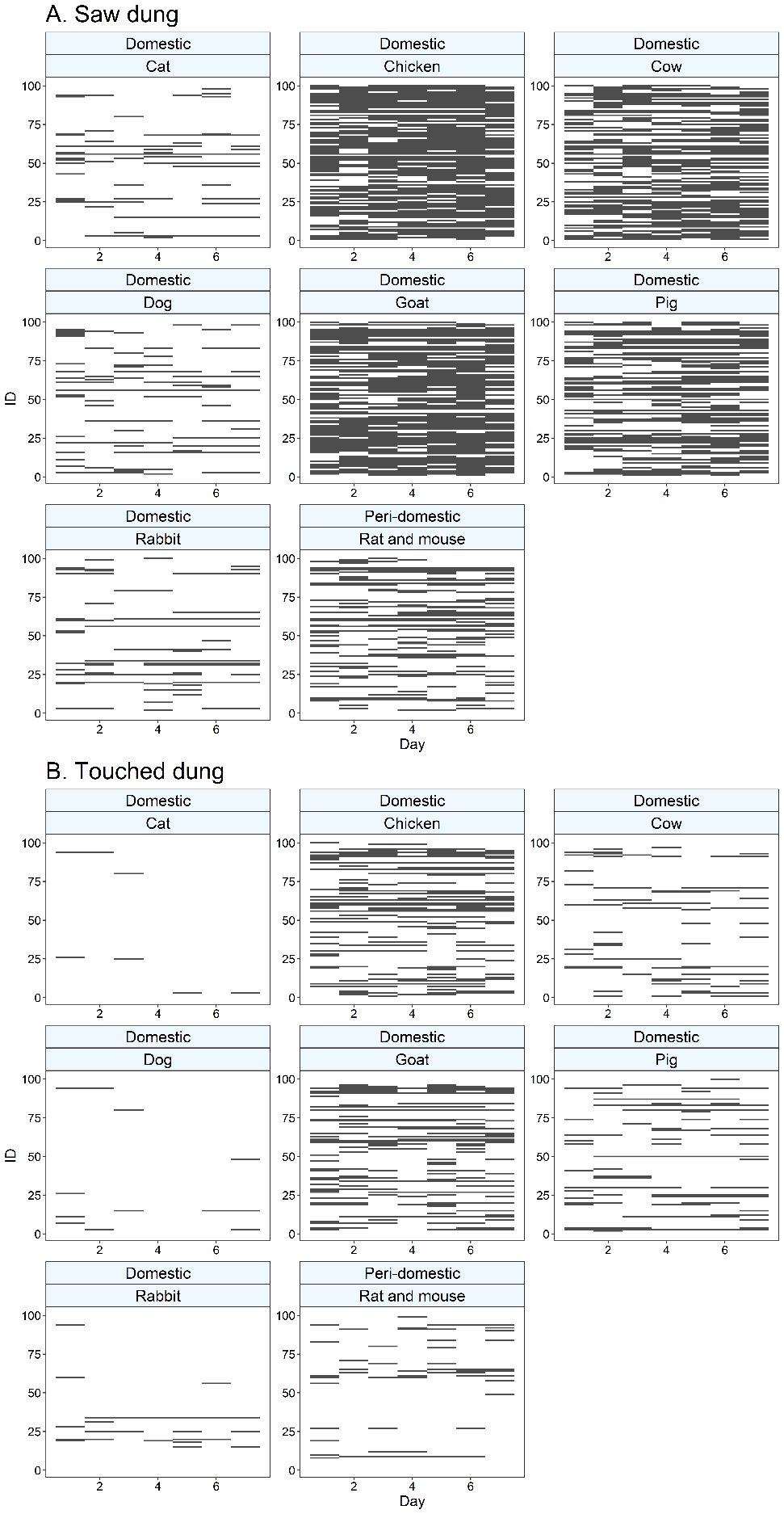


# **S7 Figure. Self-reported direct contacts between humans and animal faeces around BNP.** Contacts were reported throughout one week in a self-reported diary. A. Sightings of dung in gray. B. Direct contact with dung in gray.
