# Supplementary material for: Community health and human-animal contacts on the edges of Bwindi Impenetrable National Park, Uganda
Source: PLoS One. 2021 Nov 24;16(11):e0254467. doi: 10.1371/journal.pone.0254467 (PMC8612581; doi:10.1371/journal.pone.0254467)
Supplement: S8 Fig — Timeline with symptoms reported for a participant who reported touching a mountain gorilla. Sources for the gorilla and hand sillhouettes: Creazilla (CC BY 4.0): https://creativecommons.org/licenses/by/4.0/deed.pt; https://creazilla.com/nodes/832270-two-hands-reaching-silhouette. (DOCX) [file pone.0254467.s008.docx]

**Supporting Information**


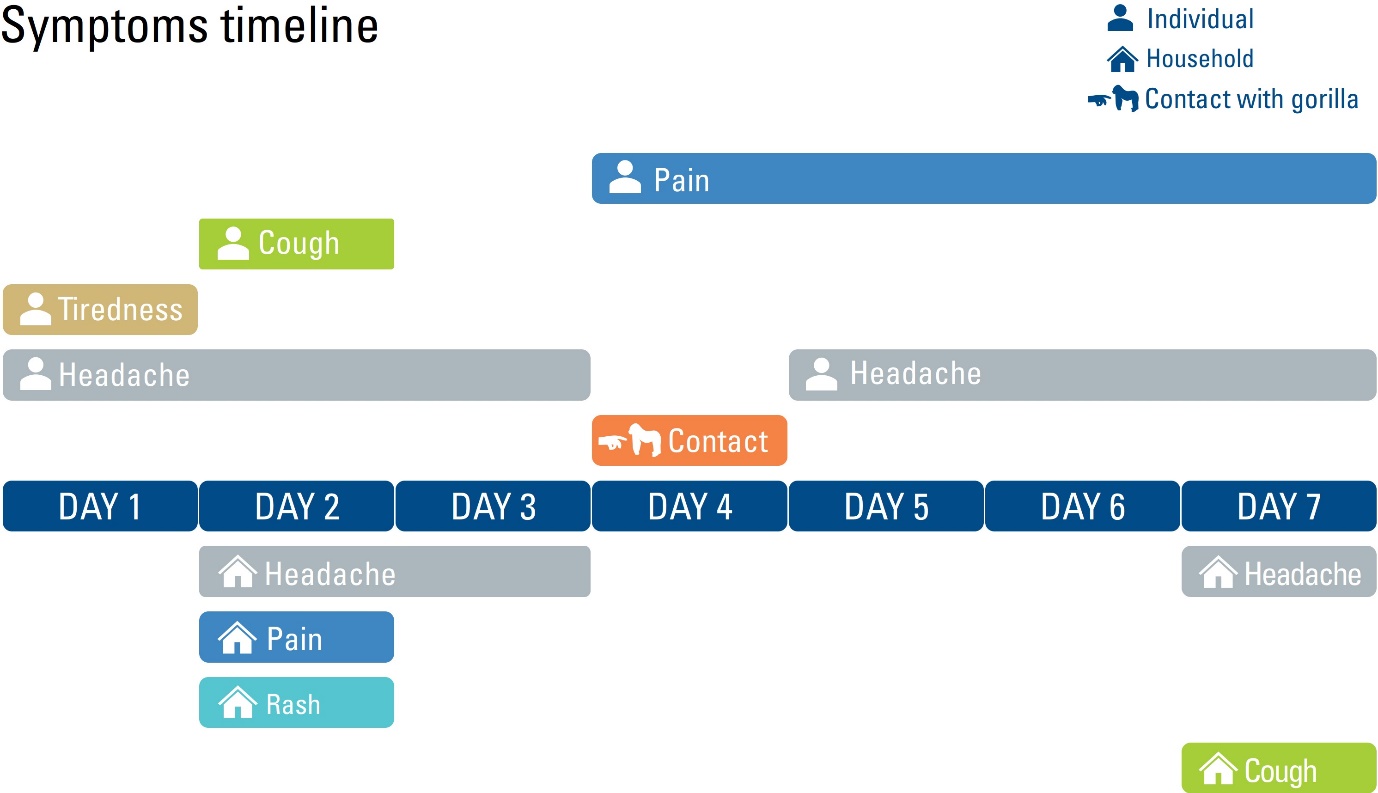


# **S8 Figure**. **Summary of self-reported information for the participant who declared to have touched a gorilla.** Timeline with symptoms reported for a participant who reported touching a mountain gorilla. Sources for the gorilla and hand sillhouettes: Creazilla (CC BY 4.0): <https://creativecommons.org/licenses/by/4.0/deed.pt>; <https://creazilla.com/nodes/832270-two-hands-reaching-silhouette>
