# Supplementary material for: Community health and human-animal contacts on the edges of Bwindi Impenetrable National Park, Uganda
Source: PLoS One. 2021 Nov 24;16(11):e0254467. doi: 10.1371/journal.pone.0254467 (PMC8612581; doi:10.1371/journal.pone.0254467)
Supplement: S1 Table — Two individuals from the total sample (n = 100) did not declare their age and were removed from the analysis. (DOCX) [file pone.0254467.s009.docx]

**Supporting Information**

**S1 Table. Age classes of self-reported direct contacts (n=98) with the people they touched during seven days in Buhoma, Uganda.** Two individuals from the total sample (n=100) did not declare their age and were removed from the analysis.

| **Age class** | **N. contacts** | **Mean** | **95% Bootstrap estimates interval** | |
| --- | --- | --- | --- | --- |
| Participant age class 16-20 interacting with 0-1 | 36 | 1.71 | 1.20 | 2.21 |
| Participant age class 16-20 interacting with 02-10 | 16 | 0.76 | 0.31 | 1.23 |
| Participant age class 16-20 interacting with 11-20 | 22 | 1.05 | 0.48 | 1.64 |
| Participant age class 16-20 interacting with 21-40 | 22 | 1.05 | 0.45 | 1.65 |
| Participant age class 16-20 interacting with 41 or more | 15 | 0.71 | 0.27 | 1.17 |
| Participant age class 21-30 interacting with 0-1 | 191 | 0.93 | 0.75 | 1.10 |
| Participant age class 21-30 interacting with 02-10 | 365 | 1.77 | 1.53 | 2.01 |
| Participant age class 21-30 interacting with 11-20 | 362 | 1.76 | 1.50 | 2.03 |
| Participant age class 21-30 interacting with 21-40 | 542 | 2.63 | 2.35 | 2.92 |
| Participant age class 21-30 interacting with 41 or more | 230 | 1.12 | 0.91 | 1.33 |
| Participant age class 31-40 interacting with 0-1 | 164 | 0.85 | 0.70 | 0.99 |
| Participant age class 31-40 interacting with 02-10 | 372 | 1.92 | 1.69 | 2.15 |
| Participant age class 31-40 interacting with 11-20 | 305 | 1.57 | 1.32 | 1.82 |
| Participant age class 31-40 interacting with 21-40 | 433 | 2.23 | 1.97 | 2.49 |
| Participant age class 31-40 interacting with 41 or more | 249 | 1.28 | 1.02 | 1.54 |
| Participant age class 41 or more interacting with 0-1 | 196 | 0.74 | 0.59 | 0.88 |
| Participant age class 41 or more interacting with 02-10 | 433 | 1.63 | 1.43 | 1.83 |
| Participant age class 41 or more interacting with 11-20 | 481 | 1.81 | 1.59 | 2.03 |
| Participant age class 41 or more interacting with 21-40 | 619 | 2.33 | 2.10 | 2.57 |
| Participant age class 41 or more interacting with 41 or more | 611 | 2.30 | 2.05 | 2.55 |
