# Supplementary material for: Community health and human-animal contacts on the edges of Bwindi Impenetrable National Park, Uganda
Source: PLoS One. 2021 Nov 24;16(11):e0254467. doi: 10.1371/journal.pone.0254467 (PMC8612581; doi:10.1371/journal.pone.0254467)
Supplement: S2 Table — Values for all species in the questionnaire are shown from highest to lowest. (DOCX) [file pone.0254467.s010.docx]

**Supporting Information**

# **S2 Table. Self-reported events of actual touching certain animals or their dung the previous week in Buhoma, Uganda (2018), according to 100 participants.** Values for all species in the questionnaire are shown from highest to lowest.

| **Taxon** | **Number of times touched along the week** |
| --- | --- |
| Chicken | 440 |
| Goat | 403 |
| Pig | 215 |
| Cow | 156 |
| Rat and Mouse | 75 |
| Rabbit | 49 |
| Dog | 48 |
| Cat | 29 |
| Monkey | 4 |
| Baboon | 0 |
| Gorilla | 1 |
| Squirrel | 2 |
| Chimpanzee | 0 |
| Civet | 1 |
| Duiker | 1 |
| Bushbuck | 0 |
| Bushpig | 0 |
| Colobus | 0 |
| Elephant | 0 |
| Porcupine | 0 |
| Total contacts | 1424 |
