# Supplementary material for: Community health and human-animal contacts on the edges of Bwindi Impenetrable National Park, Uganda
Source: PLoS One. 2021 Nov 24;16(11):e0254467. doi: 10.1371/journal.pone.0254467 (PMC8612581; doi:10.1371/journal.pone.0254467)
Supplement: S3 Table — The number of contact events is in decreasing order based on the diary week sum of events divided by seven days. (DOCX) [file pone.0254467.s011.docx]

**Supporting Information**

# **S3 Table. Link list containing the events of perception of local close contacts during a one-week survey in Buhoma, Uganda (2018).** The number of contact events is in decreasing order based on the diary week sum of events divided by seven days.

| Taxa interaction | Group | Weight (last week) | Weight (diary week/7) |
| --- | --- | --- | --- |
| Person-Person | Person-Person | 47 | 87.43 |
| Goat-Goat | Domestic-Domestic | 44 | 54.43 |
| Cow-Cow | Domestic-Domestic | 42 | 54.14 |
| Chicken-Chicken | Domestic-Domestic | 44 | 53.00 |
| Person-Cow | Domestic-Person | 6 | 44.71 |
| Pig-Pig | Domestic-Domestic | 41 | 38.43 |
| Person-Goat | Domestic-Person | 7 | 38.14 |
| Person-Chicken | Domestic-Person | 6 | 34.43 |
| Sheep-Sheep | Domestic-Domestic | 30 | 25.43 |
| Person-Pig | Domestic-Person | 5 | 24.00 |
| Dog-Dog | Domestic-Domestic | 30 | 20.00 |
| Rat and mouse-Rat and mouse | Peri-domestic-Peri-domestic | 39 | 15.29 |
| Person-Sheep | Domestic-Person | 5 | 13.43 |
| Cat-Cat | Domestic-Domestic | 23 | 12.00 |
| Rabbit-Rabbit | Domestic-Domestic | 13 | 9.86 |
| Person-Dog | Domestic-Person | 5 | 7.71 |
| Monkey-Monkey | Wild-Wild | 14 | 7.57 |
| Person-Rat and mouse | Peri-domestic-Person | 5 | 7.43 |
| Person-Rat and mouse | Peri-domestic-Person | 2 | 7.43 |
| Person-Cat | Domestic-Person | 5 | 6.71 |
| Baboon-Baboon | Wild-Wild | 18 | 6.57 |
| Gorilla-Gorilla | Wild-Wild | 10 | 5.57 |
| Person-Baboon | Wild-Person | 2 | 4.14 |
| Goat-Sheep | Domestic-Domestic | 2 | 3.57 |
| Person-Rabbit | Domestic-Person | 3 | 3.43 |
| Duiker-Duiker | Wild-Wild | 6 | 2.86 |
| Cow-Goat | Domestic-Domestic | 0 | 2.86 |
| Cow-Sheep | Domestic-Domestic | 0 | 2.71 |
| Colobus-Colobus | Wild-Wild | 8 | 2.29 |
| Squirrel-Squirrel | Wild-Wild | 5 | 2.14 |
| Elephant-Elephant | Wild-Wild | 4 | 2.14 |
| Bushpig-Bushpig | Wild-Wild | 4 | 2.14 |
| Bushbuck-Bushbuck | Wild-Wild | 4 | 2.14 |
| Chimpanzee-Chimpanzee | Wild-Wild | 6 | 2.00 |
| Person-Gorilla | Wild-Person | 3 | 1.71 |
| Person-Monkey | Wild-Person | 5 | 1.57 |
| Person-Bushpig | Wild-Person | 1 | 1.57 |
| Porcupine-Porcupine | Wild-Wild | 4 | 1.43 |
| Civet-Civet | Wild-Wild | 4 | 1.43 |
| Cat-Rat and mouse | Peri-domestic-Domestic | 0 | 1.29 |
| Goat-Chicken | Domestic-Domestic | 0 | 0.71 |
| Person-Bushbuck | Wild-Person | 1 | 0.43 |
| Dog-Chicken | Domestic-Domestic | 1 | 0.43 |
| Cow-Dog | Domestic-Domestic | 0 | 0.43 |
| Chicken-Rabbit | Domestic-Domestic | 0 | 0.43 |
| Sheep-Chicken | Domestic-Domestic | 0 | 0.29 |
| Sheep-Dog | Domestic-Domestic | 0 | 0.29 |
| Sheep-Cat | Domestic-Domestic | 0 | 0.29 |
| Pig-Dog | Domestic-Domestic | 1 | 0.29 |
| Pig-Chicken | Domestic-Domestic | 1 | 0.29 |
| Person-Squirrel | Wild-Person | 1 | 0.29 |
| Gorilla-Baboon | Wild-Wild | 0 | 0.29 |
| Goat-Dog | Domestic-Domestic | 0 | 0.29 |
| Goat-Rat and mouse | Peri-domestic-Domestic | 0 | 0.29 |
| Dog-Rat and mouse | Peri-domestic-Domestic | 1 | 0.29 |
| Dog-Rabbit | Domestic-Domestic | 0 | 0.29 |
| Cow-Chicken | Domestic-Domestic | 1 | 0.29 |
| Chicken-Rat and mouse | Peri-domestic-Domestic | 2 | 0.29 |
| Cat-Dog | Domestic-Domestic | 3 | 0.29 |
| Sheep-Pig | Domestic-Domestic | 6 | 0.14 |
| Sheep-Rat and mouse | Peri-domestic-Domestic | 0 | 0.14 |
| Rabbit-Squirrel | Wild-Domestic | 2 | 0.14 |
| Person-Duiker | Wild-Person | 0 | 0.14 |
| Person-Porcupine | Wild-Person | 0 | 0.14 |
| Monkey-Bushpig | Wild-Wild | 0 | 0.14 |
| Monkey-Baboon | Wild-Wild | 0 | 0.14 |
| Gorilla-Cow | Wild-Domestic | 0 | 0.14 |
| Gorilla-Monkey | Wild-Wild | 1 | 0.14 |
| Gorilla-Dog | Wild-Domestic | 0 | 0.14 |
| Goat-Cat | Domestic-Domestic | 0 | 0.14 |
| Elephant-Colobus | Wild-Wild | 0 | 0.14 |
| Elephant-Dog | Wild-Domestic | 0 | 0.14 |
| Cow-Pig | Domestic-Domestic | 0 | 0.14 |
| Cow-Cat | Domestic-Domestic | 1 | 0.14 |
| Chimpanzee-Colobus | Wild-Wild | 0 | 0.14 |
| Chimpanzee-Dog | Wild-Domestic | 0 | 0.14 |
| Bushpig-Pig | Wild-Domestic | 0 | 0.14 |
| Bushbuck-Cow | Wild-Domestic | 1 | 0.14 |
| Rat and mouse-Rabbit | Peri-domestic-Domestic | 1 | 0.00 |
| Porcupine-Cow | Wild-Domestic | 1 | 0.00 |
| Porcupine-Rabbit | Wild-Domestic | 1 | 0.00 |
| Porcupine-Cat | Wild-Domestic | 1 | 0.00 |
| Pig-Cat | Domestic-Domestic | 2 | 0.00 |
| Person-Colobus | Wild-Person | 1 | 0.00 |
| Person-Chimpanzee | Wild-Person | 1 | 0.00 |
| Person-Elephant | Wild-Person | 1 | 0.00 |
| Goat-Pig | Domestic-Domestic | 1 | 0.00 |
| Cow-Rabbit | Domestic-Domestic | 1 | 0.00 |
| Cow-Rat and mouse | Peri-domestic-Domestic | 1 | 0.00 |
| Cat-Chicken | Domestic-Domestic | 1 | 0.00 |
| Bushbuck-Sheep | Wild-Domestic | 1 | 0.00 |
| Baboon-Bushpig | Wild-Wild | 1 | 0.00 |
