# Supplementary material for: Community health and human-animal contacts on the edges of Bwindi Impenetrable National Park, Uganda
Source: PLoS One. 2021 Nov 24;16(11):e0254467. doi: 10.1371/journal.pone.0254467 (PMC8612581; doi:10.1371/journal.pone.0254467)
Supplement: S4 Table — Data is arranged in ascending order of the number of species a node interacted with, including the node itself. (DOCX) [file pone.0254467.s012.docx]

**Supporting Information**

# **S4 Table. Taxon-level metrics reported in Buhoma according to the participant’s observations of human and animal close contacts**. The data is arranged in ascending order of the number of species a node interacted with, including the node itself.

| Taxa | Group | Degree last week | Degree diary week |
| --- | --- | --- | --- |
| Person | Person | 20 | 18 |
| Dog | Domestic | 6 | 13 |
| Cow | Domestic | 8 | 10 |
| Sheep | Domestic | 5 | 9 |
| Chicken | Domestic | 7 | 9 |
| Goat | Domestic | 4 | 8 |
| Rat and mouse | Rat and mouse | 7 | 7 |
| Pig | Domestic | 7 | 7 |
| Cat | Domestic | 7 | 7 |
| Gorilla | Wild | 3 | 6 |
| Rabbit | Domestic | 6 | 5 |
| Monkey | Wild | 3 | 5 |
| Bushpig | Wild | 3 | 4 |
| Baboon | Wild | 3 | 4 |
| Squirrel | Wild | 3 | 3 |
| Elephant | Wild | 2 | 3 |
| Colobus | Wild | 2 | 3 |
| Chimpanzee | Wild | 2 | 3 |
| Bushbuck | Wild | 4 | 3 |
| Porcupine | Wild | 4 | 2 |
| Duiker | Wild | 1 | 2 |
| Civet | Wild | 1 | 1 |
