# Supplementary material for: Community health and human-animal contacts on the edges of Bwindi Impenetrable National Park, Uganda
Source: PLoS One. 2021 Nov 24;16(11):e0254467. doi: 10.1371/journal.pone.0254467 (PMC8612581; doi:10.1371/journal.pone.0254467)
Supplement: S1 File — (PDF) [file pone.0254467.s013.pdf]

Background questions | File number ____

1. Village name ____
2. Parish name ____
3. GPS or North/South or Latitude/Longitude __________________
4. Participant's date of birth _______________________________
5. Gender ✓
   a) Male .... ☐
   b) Female .... ☐
   c) Prefer not to answer .... ☐
6. Ethnic group ✓
   a) Bakiga .... ☐
   b) Batwa .... ☐
   c) Other:...[___________________________]...................... ☐
   d) Prefer not to answer .... ☐

File number ____

7. Education level

I. Primary level ✓

a) Complete ………………….. ☐

b) Not complete ……………. ☐

c) None ………………………. ☐

II. Secondary level

a) Senior 1 - 4 ✓

a) Complete ………………….. ☐

b) Not complete ……………. ☐

c) None ……………………….. ☐

b) Senior 5 – 6 ✓

a) Complete ………………….. ☐

b) Not complete ……………. ☐

c) None ……………………….. ☐

III. Tertiary level ✓

a) University ………………….. ☐

b) College ……………………… ☐

8. What are your main tasks at home? ________________________________

____

9. What are your main tasks when you are not at home? _________________

____

10. Do you have a latrine at home? ___________________________________

11. Is your latrine: ✓
    a) Covered................................ ☐
    b) Not covered........................ ☐

12. Do you have a bed net? _____________________

13. How many bed nets do you have? _____________

14. Are you married? ✓
    a) Yes .... ☐
    b) No .... ☐

15. What age did you marry? ________________

16. If you have children, how many and what age are they?

Age (yrs)	✓ 1	✓ 2	✓ 3	✓ 4	✓ 5 and above
a) 0-1					
b) 2-5					
c) 6-10					
d) 11-15					
e) 16-20					
f) 21-30					
g) 31-40					
h) 41 and above					

17. How many people live in your house and what is their age?

Age (yrs)	✓ 1	✓ 2	✓ 3	✓ 4	✓ 5 and above
a) 0-1					
b) 2-5					
c) 6-10					
d) 11-15					
e) 16-20					
f) 21-30					
g) 31-40					
h) 41 and above					

File number ____

19. Have you been unwell in the last week? ✓

a) Yes ………………………………………………………………… ☐

b) No ………………………………………………………………… ☐

c) Prefer not to answer …………………………………………… ☐

20. Have you been to the hospital or health center in the last week?

a) Yes ………………………………………………………………… ☐

b) No ………………………………………………………………… ☐

c) Prefer not to answer …………………………………………… ☐

21. Have you taken medical treatments in the last week?

a) Yes ………………………………………………………………… ☐

b) No ………………………………………………………………… ☐

c) Prefer not to answer …………………………………………… ☐

22. If you took medical treatments in the last week were they:

a) Self treatment from home ……………………………………… ☐

b) Given to you by a traditional healer ………………………… ☐

c) Given to you or suggested by a health worker ………… ☐

d) Prefer not to answer …………………………………………… ☐

File number ____

21. If you have been unwell in the last week, what symptoms did you have and how bad were they? (You may have had many so can have more than one)

	✓ **Mild** does not interfere with your day too much	✓ **Moderate** interferes a lot with your life, but you can be independent	✓ **Severe** you were unable to behave normally and needed substantial help
a) Tiredness	☐	☐	☐
b) Headache	☐	☐	☐
c) Fever	☐	☐	☐
d) Vomiting	☐	☐	☐
e) Diarrhea	☐	☐	☐
f) Cough	☐	☐	☐
g) Difficulty breathing	☐	☐	☐
h) Pain	☐	☐	☐
i) Rash	☐	☐	☐
j) Itching	☐	☐	☐
k) Prefer not to answer	☐	☐	☐
l) None	☐		

File number ____

22. If others in your house have been unwell in the last week, what symptoms did they have and how bad were they? (They so can have more than one)

	✓ **Mild** does not interfere with your day too much	✓ **Moderate** interferes a lot with your life, but you can be independent	✓ **Severe** you were unable to behave normally and needed substantial help
a) Tiredness ……………………..			
b) Headache …………………….			
c) Fever …………………………..			
d) Vomiting …………………….			
e) Diarrhea …………………….			
f) Cough ………………………..			
g) Difficulty breathing ………			
h) Pain ……………………………			
i) Rash …………………………..			
j) Itching ………………………..			
k) Prefer not to answer ……			

l) None ………………………….. ☐

File number ____

23. If you have <u>ever</u> had these diseases?

		✓ Had the disease	✓ Do not know	✓ Prefer not to say
a)	Measles …………………………………………………………………	☐	☐	☐
b)	Mumps ………………………………………………………………	☐	☐	☐
c)	Rubella / German measles………………………………………	☐	☐	☐
d)	Tb / tuberculosis …………………………………………………	☐	☐	☐
e)	Polio ………………………………………………………………………	☐	☐	☐
f)	Diptheria …………………………………………………………………	☐	☐	☐
g)	Tetanus …………………………………………………………………	☐	☐	☐
h)	Whooping cough …………………………………………………	☐	☐	☐
i)	Swollen neck glands (unknown cause) ……………………	☐	☐	☐
j)	Croup / severe cough (unknown cause) …………………	☐	☐	☐
k)	Ear infection …………………………………………………………	☐	☐	☐
l)	Sinus infection ……………………………………………………	☐	☐	☐
m)	Meningitis ………………………………………………………………	☐	☐	☐
n)	Jaundice …………………………………………………………………	☐	☐	☐
f)	Ear infection …………………………………………………………	☐	☐	☐
g)	Warts (skin or genital) ………………………………………	☐	☐	☐
h)	Smallpox …………………………………………………………………	☐	☐	☐
i)	Chickenpox / Shingles …………………………………………	☐	☐	☐
j)	Other .. [________________________________]…………	☐	☐	☐
k)	Other .. [________________________________]…………	☐	☐	☐
l)	None ………………………………………………………………………	☐		

File number ____

24. Have you ever been vaccinated against these diseases and did you complete the course?

		✓ **Vaccinated** Completed	✓ **Vaccinated** Not completed	✓ **Do not know**	✓ **Not observed**
a)	Measles / MCV / MMR / MMRV ……………….	☐	☐	☐	☐
b)	Mumps / MMR / MMRV ………………………….	☐	☐	☐	☐
c)	Rubella / MR / MMR / MMRV………………….	☐	☐	☐	☐
d)	Tb / BCG ………………………………………………	☐	☐	☐	☐
e)	Polio / IPV …………………………………………….	☐	☐	☐	☐
f)	Diptheria-tetanus-pertussis / DPT ……………	☐	☐	☐	☐
g)	Diptheria-tetanus-pertussis-hepatitis B-haemophilus / DTP-HBV-HIB .	☐	☐	☐	☐
f)	Pneumococcal / PCV ……………………………….	☐	☐	☐	☐
g)	Human papillomavirus / HPV …………………..	☐	☐	☐	☐
h)	Tetanus / tt / other tetanus…………………….	☐	☐	☐	☐
i)	Smallpox / vaccinia (do you have a scar) …..	☐	☐	☐	☐
j)	Chickenpox / VZV / MMRV ………………………	☐	☐	☐	☐
k)	Rabies …………………………………………………….	☐	☐	☐	☐
l)	Other .. [_________________________]…….	☐	☐	☐	☐
m)	Other .. [_________________________]…….	☐	☐	☐	☐

File number ____

25. Which vaccine doses were given to you and at what date (from the card)?

		✓ Dose 1	Mo/Yr	✓ Dose 2	Mo/Yr	✓ Dose 3	Mo/Yr
a)	Measles / MCV / MMR / MMRV ……………….						
b)	Mumps / MMR / MMRV ………………………….						
c)	Rubella / MR / MMR / MMRV…………………..						
d)	Tb / BCG ………………………………………………..						
e)	Polio / IPV …………………………………………….						
f)	Diptheria-tetanus-pertussis / DPT …………….						
g)	Diptheria-tetanus-pertussis-hepatitis B-haemophilus / DTP-HBV-HIB .						
f)	Pneumococcal / PCV ……………………………….						
g)	Human papillomavirus / HPV …………………..						
h)	Tetanus / tt / other tetanus………………………						
i)	Smallpox / vaccinia (do you have a scar) …..						
j)	Chickenpox / VZV / MMRV ………………………						
k)	Rabies …………………………………………………..						
l)	Other .. [_________________________]……..						
m)	Other .. [_________________________]……..						

File number ____

26. Which vaccines have people living with you had (from their card)?

Person #1 : Date of birth _____

	✓ Dose 1	Mo/Yr	✓ Dose 2	Mo/Yr	✓ Dose 3	Mo/Yr
a) Measles / MCV / MMR / MMRV						
b) Mumps / MMR / MMRV						
c) Rubella / MR / MMR / MMRV......................						
d) Tb / BCG						
e) Polio / IPV						
f) Diptheria-tetanus-pertussis / DPT						
g) Diptheria-tetanus-pertussis-hepatitis B-haemophilus / DTP-HBV-HIB .						
f) Pneumococcal / PCV						
g) Human papillomavirus / HPV						
h) Tetanus / tt / other tetanus..........................						
i) Smallpox / vaccinia (do you have a scar)						
j) Chickenpox / VZV / MMRV						
k) Rabies						
l) Other .. [___________________________]........						
m) Other .. [___________________________]........						

File number ____

26. Which vaccines have people living with you had (from their card)?

Person #2 : Date of birth _____

		✓ Dose 1	Mo/Yr	✓ Dose 2	Mo/Yr	✓ Dose 3	Mo/Yr
a)	Measles / MCV / MMR / MMRV						
b)	Mumps / MMR / MMRV						
c)	Rubella / MR / MMR / MMRV......................						
d)	Tb / BCG						
e)	Polio / IPV						
f)	Diptheria-tetanus-pertussis / DPT						
g)	Diptheria-tetanus-pertussis-hepatitis B-haemophilus / DTP-HBV-HIB .						
f)	Pneumococcal / PCV						
g)	Human papillomavirus / HPV						
h)	Tetanus / tt / other tetanus.........................						
i)	Smallpox / vaccinia (do you have a scar)						
j)	Chickenpox / VZV / MMRV						
k)	Rabies						
l)	Other .. [___________________________]........						
m)	Other .. [___________________________]........						

File number ____

26. Which vaccines have people living with you had (from their card)?

Person #3 : Date of birth _____

	✓ Dose 1	Mo/Yr	✓ Dose 2	Mo/Yr	✓ Dose 3	Mo/Yr
a) Measles / MCV / MMR / MMRV						
b) Mumps / MMR / MMRV						
c) Rubella / MR / MMR / MMRV......................						
d) Tb / BCG						
e) Polio / IPV						
f) Diptheria-tetanus-pertussis / DPT						
g) Diptheria-tetanus-pertussis-hepatitis B-haemophilus / DTP-HBV-HIB .						
f) Pneumococcal / PCV						
g) Human papillomavirus / HPV						
h) Tetanus / tt / other tetanus.........................						
i) Smallpox / vaccinia (do you have a scar)						
j) Chickenpox / VZV / MMRV						
k) Rabies						
l) Other .. [___________________________]........						
m) Other .. [___________________________]........						

File number ____

26. Which vaccines have people living with you had (from their card)?

Person #4 : Date of birth _____

	✓ Dose 1	Mo/Yr	✓ Dose 2	Mo/Yr	✓ Dose 3	Mo/Yr
a) Measles / MCV / MMR / MMRV						
b) Mumps / MMR / MMRV						
c) Rubella / MR / MMR / MMRV......................						
d) Tb / BCG						
e) Polio / IPV						
f) Diptheria-tetanus-pertussis / DPT						
g) Diptheria-tetanus-pertussis-hepatitis B-haemophilus / DTP-HBV-HIB .						
f) Pneumococcal / PCV						
g) Human papillomavirus / HPV						
h) Tetanus / tt / other tetanus..........................						
i) Smallpox / vaccinia (do you have a scar)						
j) Chickenpox / VZV / MMRV						
k) Rabies						
l) Other .. [_____________________________]........						
m) Other .. [_____________________________]........						

File number ____

26. Which vaccines have people living with you had (from their card)?

Person #5 : Date of birth _____

	✓ Dose 1	Mo/Yr	✓ Dose 2	Mo/Yr	✓ Dose 3	Mo/Yr
a) Measles / MCV / MMR / MMRV						
b) Mumps / MMR / MMRV						
c) Rubella / MR / MMR / MMRV......................						
d) Tb / BCG						
e) Polio / IPV						
f) Diptheria-tetanus-pertussis / DPT						
g) Diptheria-tetanus-pertussis-hepatitis B-haemophilus / DTP-HBV-HIB .						
f) Pneumococcal / PCV						
g) Human papillomavirus / HPV						
h) Tetanus / tt / other tetanus..........................						
i) Smallpox / vaccinia (do you have a scar)						
j) Chickenpox / VZV / MMRV						
k) Rabies						
l) Other .. [___________________________].......						
m) Other .. [___________________________].......						

File number ____

26. Which vaccines have people living with you had (from their card)?

Person #6 : Date of birth _____

		✓ Dose 1	Mo/Yr	✓ Dose 2	Mo/Yr	✓ Dose 3	Mo/Yr
a)	Measles / MCV / MMR / MMRV ……………….						
b)	Mumps / MMR / MMRV ………………………….						
c)	Rubella / MR / MMR / MMRV…………………..						
d)	Tb / BCG …………………………………………………..						
e)	Polio / IPV ……………………………………………..						
f)	Diptheria-tetanus-pertussis / DPT …………….						
g)	Diptheria-tetanus-pertussis-hepatitis B-haemophilus / DTP-HBV-HIB .						
f)	Pneumococcal / PCV ……………………………….						
g)	Human papillomavirus / HPV ……………………						
h)	Tetanus / tt / other tetanus………………………						
i)	Smallpox / vaccinia (do you have a scar) …..						
j)	Chickenpox / VZV / MMRV ………………………						
k)	Rabies …………………………………………………….						
l)	Other .. [___________________________]……..						
m)	Other .. [___________________________]……..						

File number ____

26. Which vaccines have people living with you had (from their card)?

Person #7 : Date of birth _____

	✓ Dose 1	Mo/Yr	✓ Dose 2	Mo/Yr	✓ Dose 3	Mo/Yr
a) Measles / MCV / MMR / MMRV						
b) Mumps / MMR / MMRV						
c) Rubella / MR / MMR / MMRV......................						
d) Tb / BCG						
e) Polio / IPV						
f) Diptheria-tetanus-pertussis / DPT						
g) Diptheria-tetanus-pertussis-hepatitis B-haemophilus / DTP-HBV-HIB .						
f) Pneumococcal / PCV						
g) Human papillomavirus / HPV						
h) Tetanus / tt / other tetanus..........................						
i) Smallpox / vaccinia (do you have a scar)						
j) Chickenpox / VZV / MMRV						
k) Rabies						
l) Other .. [_________________________]........						
m) Other .. [_________________________]........						

File number ____

26. Which vaccines have people living with you had (from their card)?

Person #8 : Date of birth _____

		✓ **Dose 1**	**Mo/Yr**	✓ **Dose 2**	**Mo/Yr**	✓ **Dose 3**	**Mo/Yr**
a)	Measles / MCV / MMR / MMRV						
b)	Mumps / MMR / MMRV						
c)	Rubella / MR / MMR / MMRV.......................						
d)	Tb / BCG						
e)	Polio / IPV						
f)	Diptheria-tetanus-pertussis / DPT						
g)	Diptheria-tetanus-pertussis-hepatitis B-haemophilus / DTP-HBV-HIB .						
f)	Pneumococcal / PCV						
g)	Human papillomavirus / HPV						
h)	Tetanus / tt / other tetanus..........................						
i)	Smallpox / vaccinia (do you have a scar)						
j)	Chickenpox / VZV / MMRV						
k)	Rabies						
l)	Other .. [__________________________]........						
m)	Other .. [__________________________]........						

27. In the last week, have you seen, touched or got close (like in the same field, trees or plantation) to the following: (not grey, ✓ each pair)

	Person	Gorilla	Monkey	Baboon	Bushpig	Civet	Chimpanzee	Elephant	Colobus	Porcupine	Duiker	Bushbuck	Cow	Goat	Sheep	Pig	Cat	Dog	Chicken	Rat	Mouse	Rabbit	Squirrel
Person																							
Gorilla																							
Monkey																							
Baboon																							
Bushpig																							
Civet																							
Chimpanzee																							
Elephant																							
Colobus																							
Porcupine																							
Duiker																							
Bushbuck																							
Cow																							
Goat																							
Sheep																							
Pig																							
Cat																							
Dog																							
Chicken																							
Rat																							
Mouse																							
Rabbit																							
Squirrel																							

28. Have you <u>ever</u> seen, touched or got close (like in the same field, trees or plantation) to the following: (not grey, ✓ each pair)

	Person	Gorilla	Monkey	Baboon	Bushpig	Civet	Chimpanzee	Elephant	Colobus	Porcupine	Duiker	Bushbuck	Cow	Goat	Sheep	Pig	Cat	Dog	Chicken	Rat	Mouse	Rabbit	Squirrel
Person																							
Gorilla																							
Monkey																							
Baboon																							
Bushpig																							
Civet																							
Chimpanzee																							
Elephant																							
Colobus																							
Porcupine																							
Duiker																							
Bushbuck																							
Cow																							
Goat																							
Sheep																							
Pig																							
Cat																							
Dog																							
Chicken																							
Rat																							
Mouse																							
Rabbit																							
Squirrel																							
