# Supplementary material for: Community health and human-animal contacts on the edges of Bwindi Impenetrable National Park, Uganda
Source: PLoS One. 2021 Nov 24;16(11):e0254467. doi: 10.1371/journal.pone.0254467 (PMC8612581; doi:10.1371/journal.pone.0254467)

Diary questions

File number ________ | Day ___ of 7

1. Did you sleep under a bed net last night?

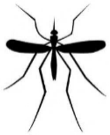
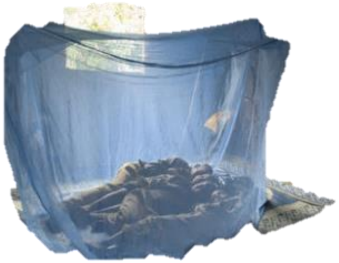

✓

a) Yes ……………………………. ☐
b) No ……………………………... ☐
c) Prefer not to answer … ☐

2. Did you attend a meeting with any group of people at home, work or in the community today?

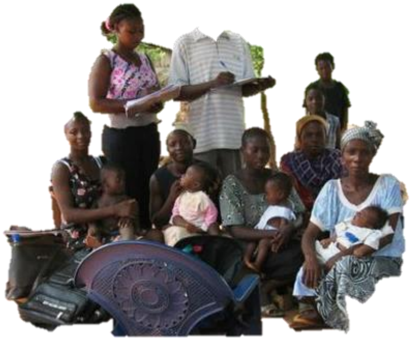

✓

a) Yes ……………………………. ☐
b) No ……………………………. ☐
c) Prefer not to answer … ☐

File number ________ | Day ___ of 7

3. Did you touch other people today in any way?

This can include handshakes, picking up children, sleeping in the same bed, or any physical contact

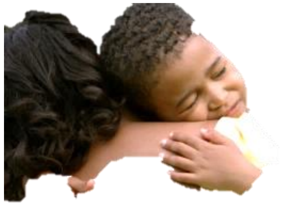

✓

a) Yes .... ☐
b) No .... ☐
c) Prefer not to answer ... ☐

4. Did you see other people touch each other in your house today in any way?

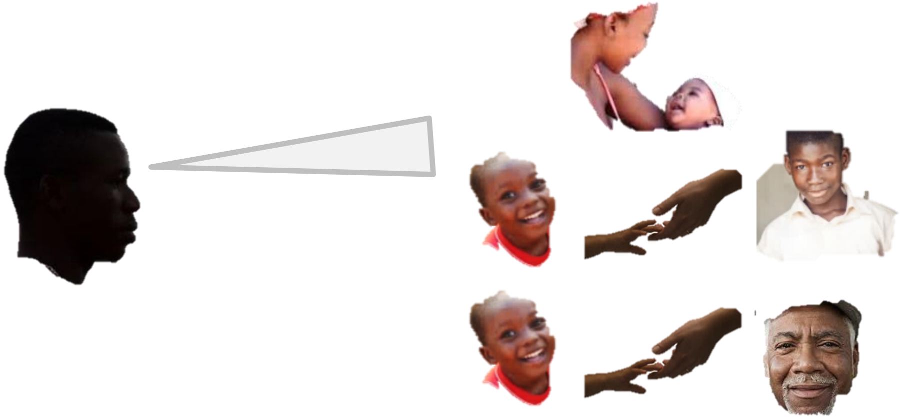

✓

a) Yes .... ☐
b) No .... ☐
c) Prefer not to answer ... ☐

File number ________ | Day ___ of 7

5. If <u>you touched people today</u>, what age and how many people of that age you touched today?

	Age ↓	Number of people ↓ 1	2	3	4	5 and above
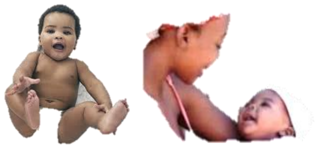	a) 0-1 years					
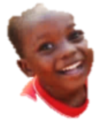	b) 2-10 years					
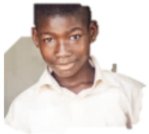	c) 11-20 years					
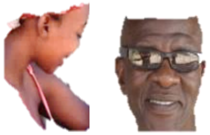	d) 21-40 years					
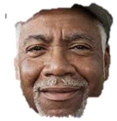	e) 41 and above					

File number ________ | Day ___ of 7

6. If you saw others touching <u>today</u>, what age people were touching?

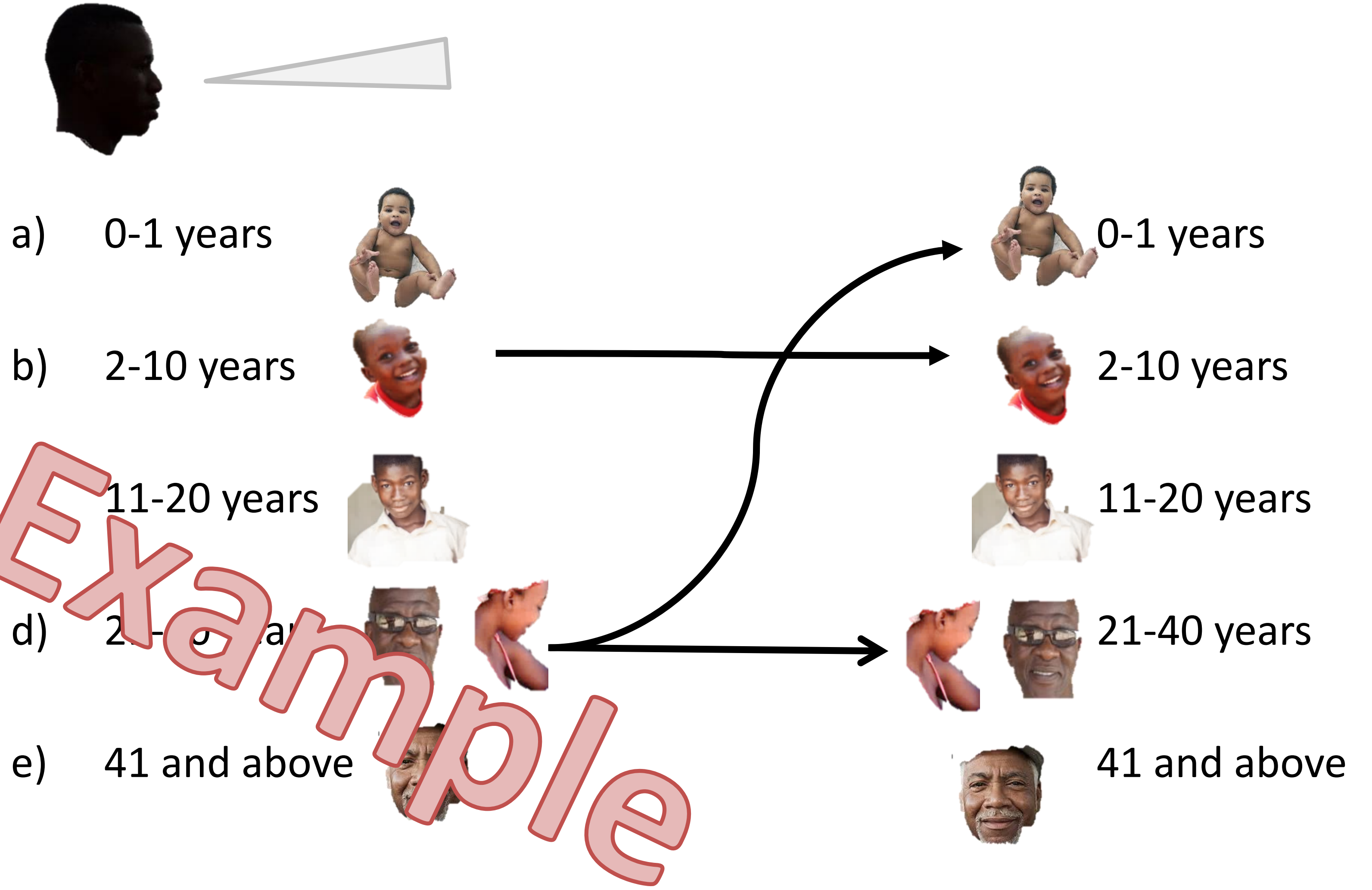

File number ________ | Day ___ of 7

6. If you saw others touching <u>today</u>, what age people were touching?

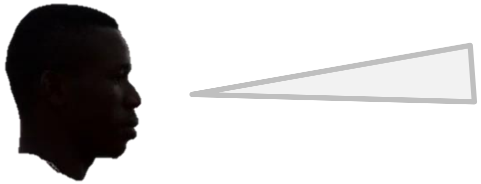

a) 0-1 years 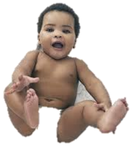

b) 2-10 years 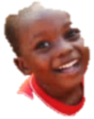

c) 11-20 years 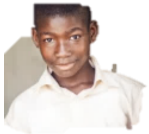

d) 21-40 years 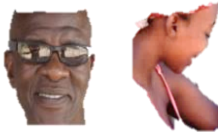

e) 41 and above 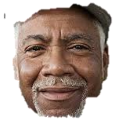

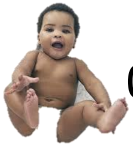 0-1 years

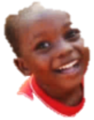 2-10 years

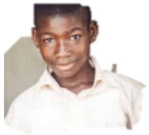 11-20 years

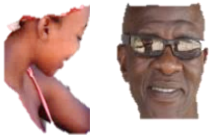 21-40 years

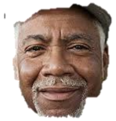 41 and above

File number ________ | Day ___ of 7

7. Did you use the pit latrine or toilet today and how many times?

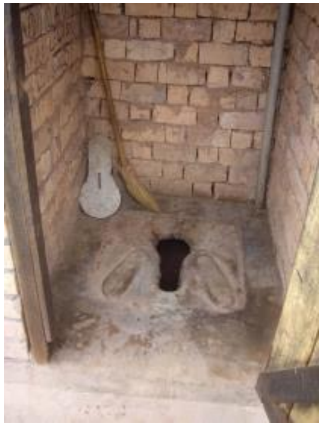

	✓
0	
1	
2	
3	
4	
5 and above	
Prefer not to answer	

8. Did you use toilet paper or any other anal cleansing material when you went today?

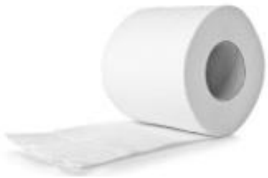

	✓
a) Yes	
b) Sometimes	
c) No	
d) Prefer not to answer ...	

File number ________ | Day ___ of 7

9. Did you wash your hands after the toilet today and how many times?

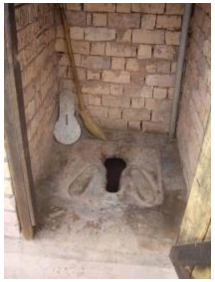

→

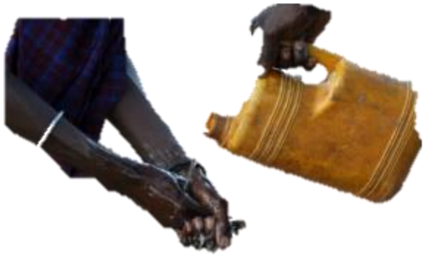

✓

a) Yes ………………………… ☐
b) Sometimes ……………… ☐
c) No …………………………… ☐
d) Prefer not to answer … ☐

10. Did you use soap to wash your hands after the toilet today and how many times?

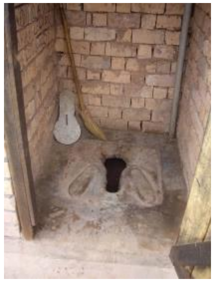

→

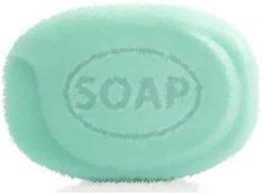

✓

a) Yes ………………………… ☐
b) Sometimes ……………… ☐
c) No …………………………… ☐
d) Prefer not to answer … ☐

File number ________ | Day ___ of 7

11. Where did you wash your hands after the toilet today?

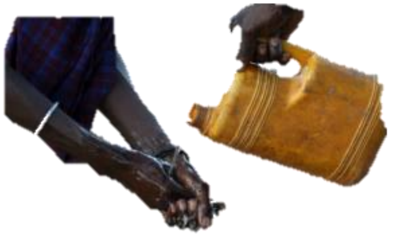

?

✓

a) In the house ……………..
b) Near the toilet ………….
c) Somewhere else ……….
d) Prefer not to answer ….

12. Did you prepare food today?

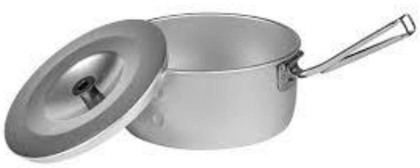

✓

a) Yes ……………………………
b) No ……………………………

File number ________ | Day ___ of 7

13. How many times did you prepare food today?

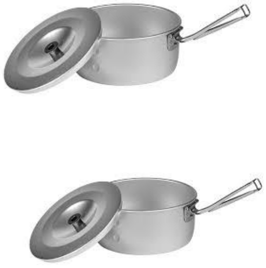

?

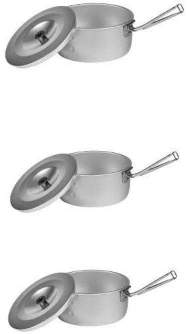

Option	✓
0	☐
1	☐
2	☐
3	☐
4	☐
5 and above	☐
Prefer not to answer	☐

14. Did you wash your hands before preparing food today and how many times?

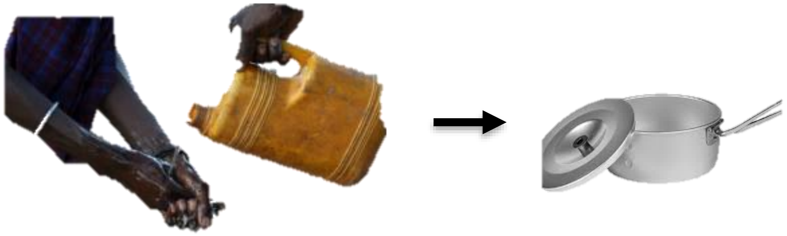

Option	✓
a) Yes	☐
b) Sometimes	☐
c) No	☐
d) Prefer not to answer …	☐

File number ________ | Day ___ of 7

15. Did you wash your hands after preparing food today and how many times?

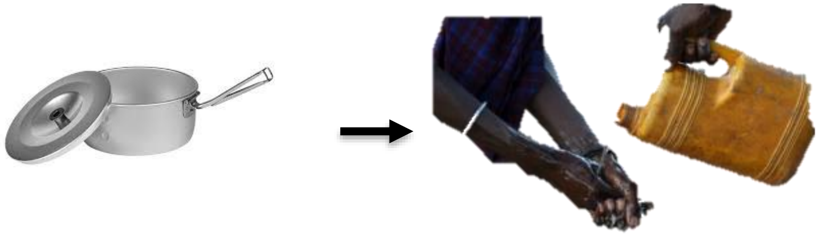

	✓
a) Yes ……………………………….	
b) Sometimes ……………….	
c) No …………………………….	
d) Prefer not to answer …	

16. Did you use soap to wash your hands when preparing food today and how many times?

?

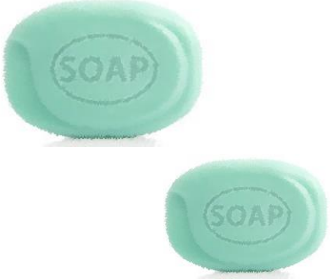

	✓
a) Yes ……………………………….	
b) Sometimes ……………….	
c) No …………………………….	
d) Prefer not to answer …	

17. Did you prepare meat today?

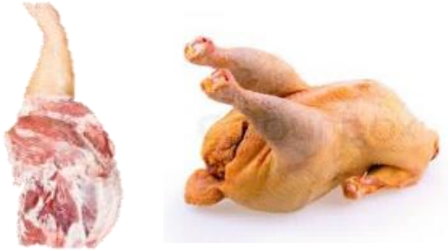

✓

a) Yes ……………………………… ☐
b) No ……………………………… ☐
c) Prefer not to answer … ☐

18. Did you touch animal blood today?

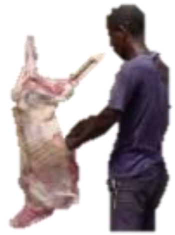

✓

a) Yes ……………………………… ☐
b) No ……………………………… ☐
c) Prefer not to answer … ☐

19. Did you get bitten or scratched today?

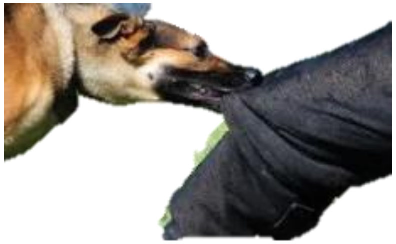

✓

a) Yes ……………………………… ☐
b) No ……………………………… ☐
c) Prefer not to answer … ☐

File number ________ | Day ___ of 7

20. Did you get cut today?

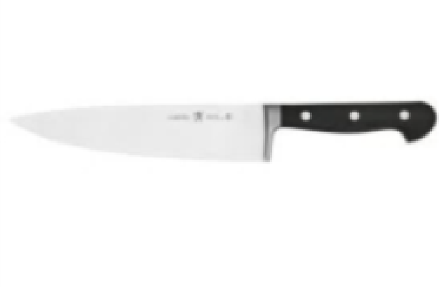

		✓
a)	Yes	
b)	No	
c)	Prefer not to answer ...	

21. If yes, did you wash if you got cut, bitten or scratched your hands afterward

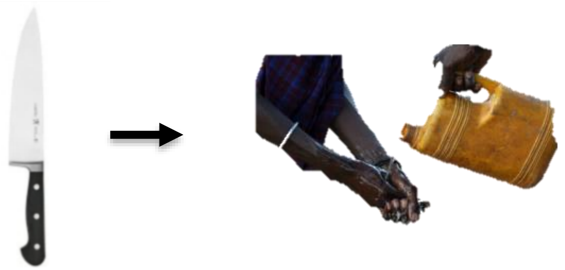

		✓
a)	Yes	
b)	No	
c)	Prefer not to answer ...	

22. Did you use soap to wash your hands after getting cut, bitten or scratched?

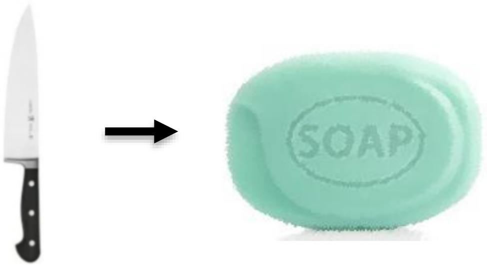

		✓
a)	Yes	
b)	No	
c)	Prefer not to answer ...	

File number ________ | Day ___ of 7

23. Did you eat today? ✓

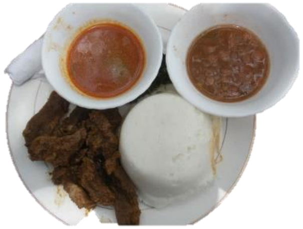

a) Yes ....
b) No ....
c) Prefer not to answer ...

24. How many times did you eat today? ✓

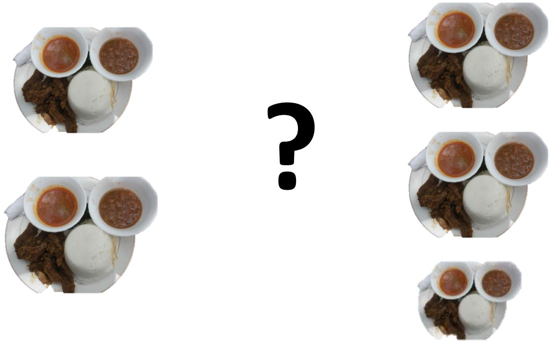

0 ....
1 ....
2 ....
3 ....
4 and above ....
Prefer not to answer ....

25. Did you cover any leftover food today? ✓

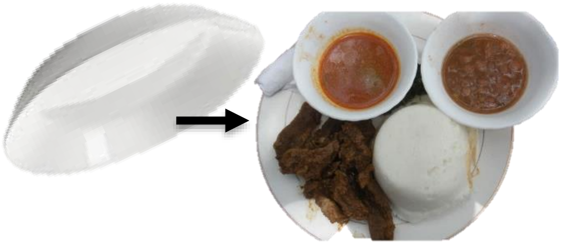

a) Yes ....
b) Sometimes ....
c) No ....
d) Prefer not to answer ...

File number _________ | Day ___ of 7

26. Did you wash your hands before eating food today and how many times?

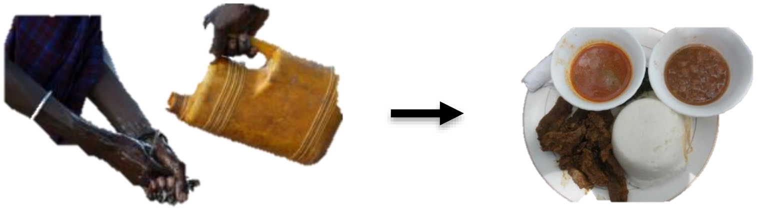

✓

a) Yes ……………………………

b) Sometimes ……………….

c) No ……………………………

d) Prefer not to answer …

27. Did you use utensils to eat your food today and how many times?

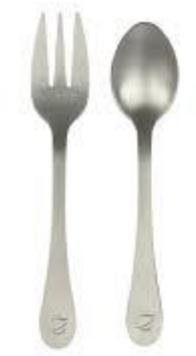

✓

a) Yes ……………………………

b) Sometimes ……………….

c) No ……………………………

d) Prefer not to answer …

File number ________ | Day ___ of 7

28. Did you use soap to wash your hands at mealtimes today and how many times?

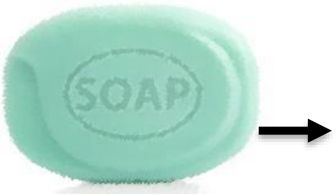

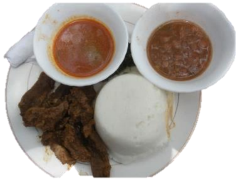

✓

a) Yes ………………………………… ☐
b) Sometimes ………………. ☐
c) No ……………………………… ☐
d) Prefer not to answer … ☐

29. Did you use a drying rack to dry your utensils today and how many times?

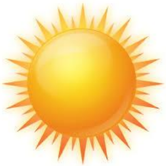
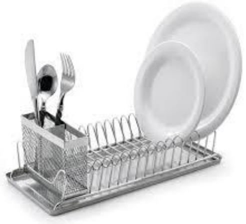

✓

a) Yes ………………………………… ☐
b) Sometimes ………………. ☐
c) No ……………………………… ☐
d) Prefer not to answer … ☐

File number _________ | Day ___ of 7

30. Did you eat or drink the following food today? ✓

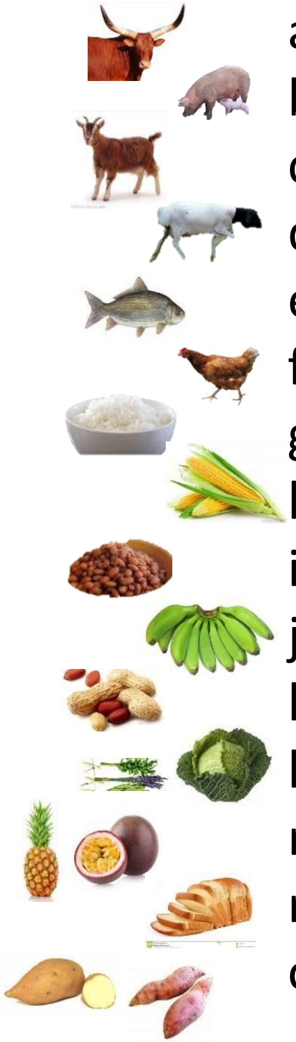

a) Beef ....
b) Pork ....
c) Goat ....
d) Other meat ....
e) Fish ....
f) Chicken ....
g) Rice ....
h) Posho ....
i) Beans ....
j) Matooke ....
k) Groundnut ....
l) Vegetables ....
m) Fruit ....
n) Bread ....
o) Sweet potato ....

30. (Continued): Did you eat or drink the following food today?

		✓
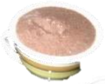 a)	Porridge	
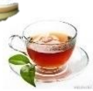 b)	Tea	
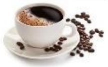 c)	Coffee	
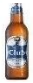 d)	Beer	
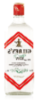 e)	Gin or other spirit	
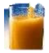 f)	Fruit juice	
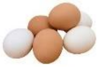 g)	Eggs	
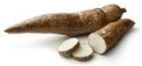 h)	Cassava	
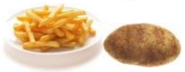 i)	Irish potato	

File number ________ | Day ___ of 7

31. Did you prepare drinks today with <u>local</u> water today?

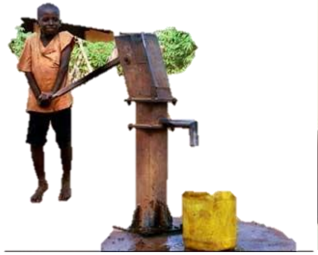
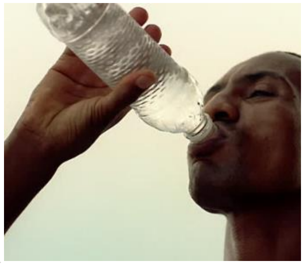
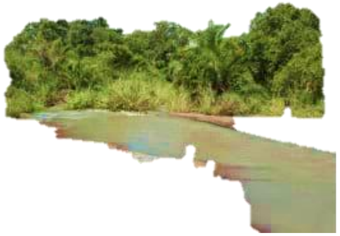

✓

a) Yes ………………………………
b) No ………………………………
c) Prefer not to answer …

32. Did you boil or filter the water today?

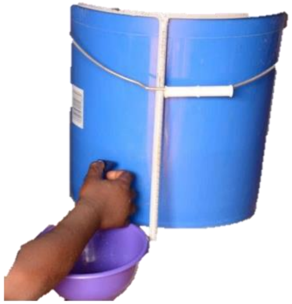
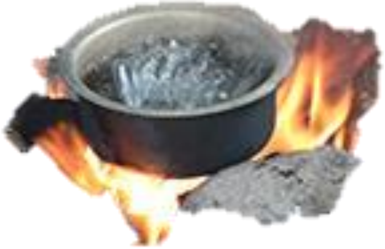

✓

a) Yes ………………………………
b) No ………………………………
c) Prefer not to answer …

File number ________ | Day ___ of 7

33. Did you drink milk today?

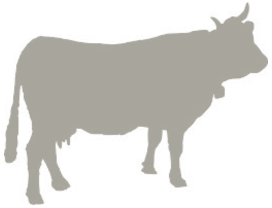

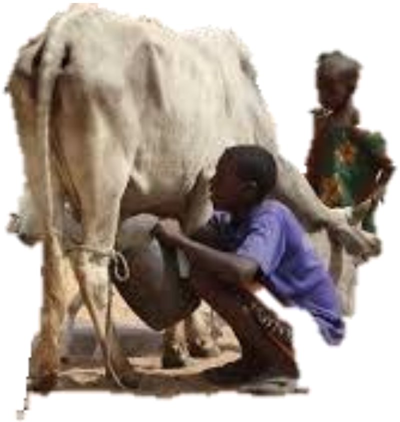

✓

a) Yes ……………………………. ☐
b) No ……………………………. ☐
c) Prefer not to answer … ☐

34. If so, did you boil the milk you drank today?

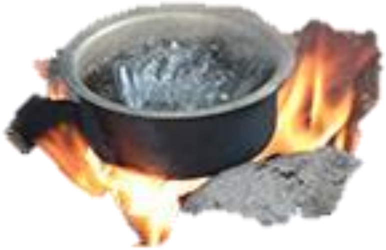

✓

a) Yes ……………………………. ☐
b) No ……………………………. ☐
c) Prefer not to answer … ☐

File number __________ | Day ____ of 7

35. Today, have you been unwell, what symptoms did they have and how bad were they? (You can have more than one)

	✓ **Mild** does not interfere with your day too much	✓ **Moderate** interferes a lot with your life, but you can be independent	✓ **Severe** you were unable to behave normally and needed substantial help
a) Tiredness			
b) Headache			
c) Fever			
d) Vomiting			
e) Diarrhea			
f) Cough			
g) Difficulty breathing			
h) Pain			
i) Rash			
j) Itching			
k) Other ...[____________].....			
l) Prefer not to answer			

m) None ☐

File number __________ | Day ____ of 7

36. Today, has <u>anyone</u> in your house been unwell, what symptoms did they have and how bad were they? (They can have more than one)

	✓ **Mild** does not interfere with your day too much	✓ **Moderate** interferes a lot with your life, but you can be independent	✓ **Severe** you were unable to behave normally and needed substantial help
a) Tiredness	☐	☐	☐
b) Headache	☐	☐	☐
c) Fever	☐	☐	☐
d) Vomiting	☐	☐	☐
e) Diarrhea	☐	☐	☐
f) Cough	☐	☐	☐
g) Difficulty breathing	☐	☐	☐
h) Pain	☐	☐	☐
i) Rash	☐	☐	☐
j) Itching	☐	☐	☐
k) Other …[____________]…..	☐	☐	☐
l) Prefer not to answer ……	☐	☐	☐
m) None	☐		

File number __________ | Day ____ of 7

37. Did you visit the clinic or hospital today?

	✓
a) Yes	
b) No	
c) Prefer not to answer ..	

38. Did you take medicine from the hospital today?

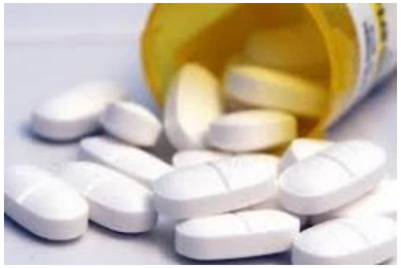

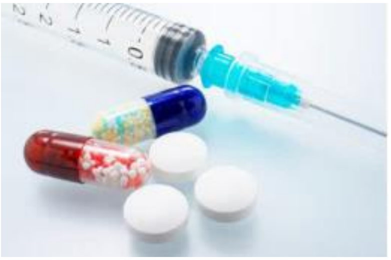

	✓
a) Yes	
b) No	
c) Prefer not to answer ..	

File number __________ | Day ___ of 7

39. Did you visit a traditional healer today?

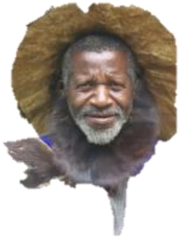

✓

a) Yes .... ☐
b) No .... ☐
c) Prefer not to answer .. ☐

40. Did you take medicine from the traditional healer today?

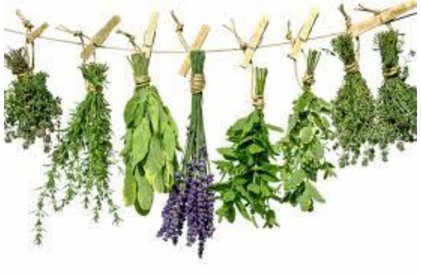

✓

a) Yes .... ☐
b) No .... ☐
c) Prefer not to answer .. ☐

41. Did you take medicines you prepared at home today?

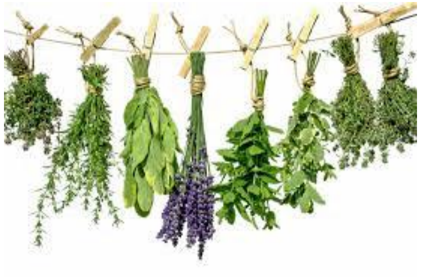

✓

a) Yes .... ☐
b) No .... ☐
c) Prefer not to answer .. ☐

File number ________ | Day ___ of 7

42. Did you see around your house or touch these animals today:

✓ ✓

a) Cows 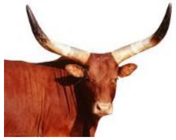 ------------------- 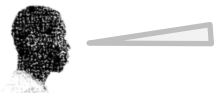 ☐ 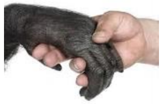 ☐

b) Goats 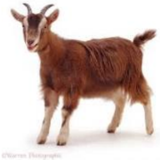 ------------------- 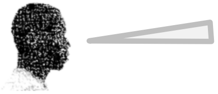 ☐ 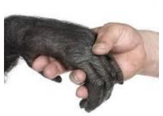 ☐

c) Pig 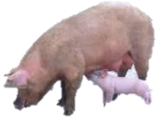 ------------------- 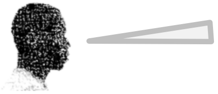 ☐ 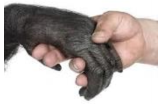 ☐

d) Chicken 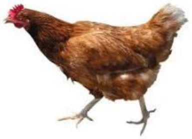 ------------------- 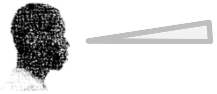 ☐ 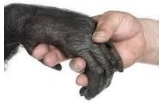 ☐

e) Rats 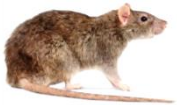 ------------------- 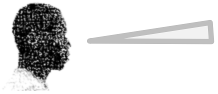 ☐ 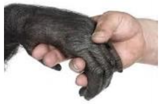 ☐

f) Rabbits 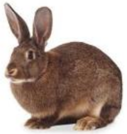 ------------------- 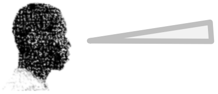 ☐ 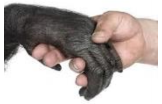 ☐

File number ________ | Day ___ of 7

43. Did you see or touch any animal dung from these animals today:

a) Cows

b) Goats

c) Pig

d) Chicken

e) Rats

f) Rabbits

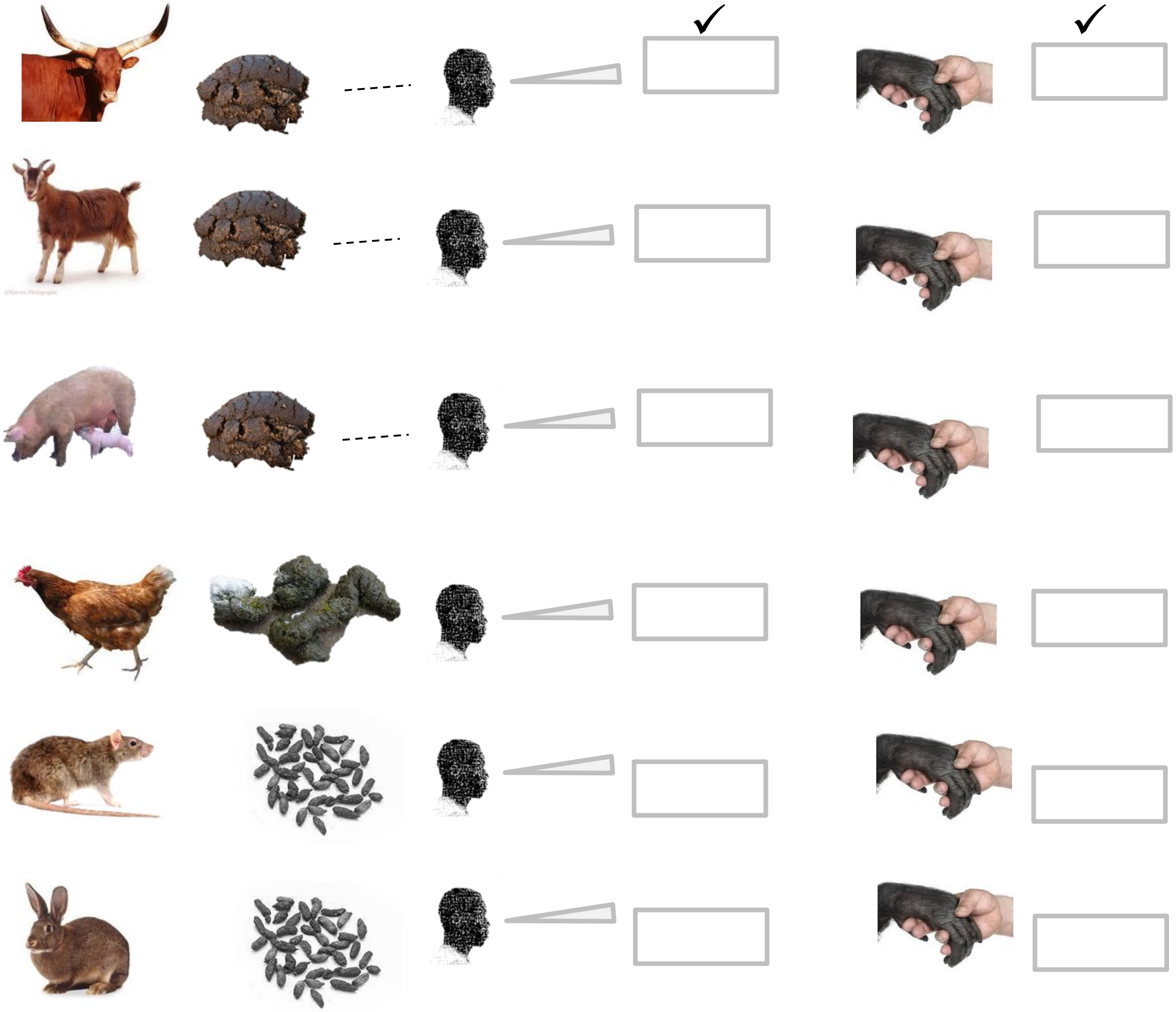

File number ________ | Day ___ of 7

44. Did you see or touch any animals or dung from these animals today:

a) Cat

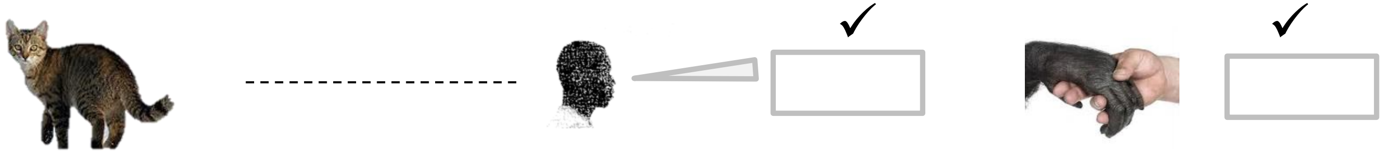

b) Dog

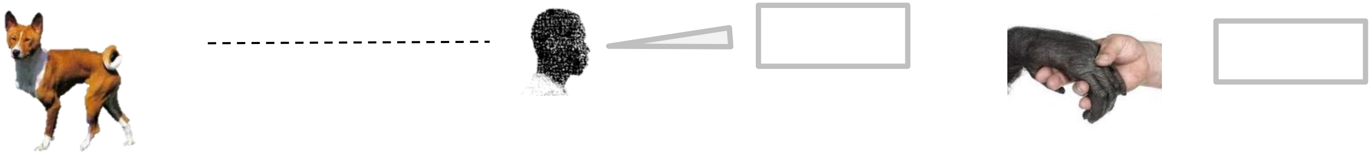

c) Cat

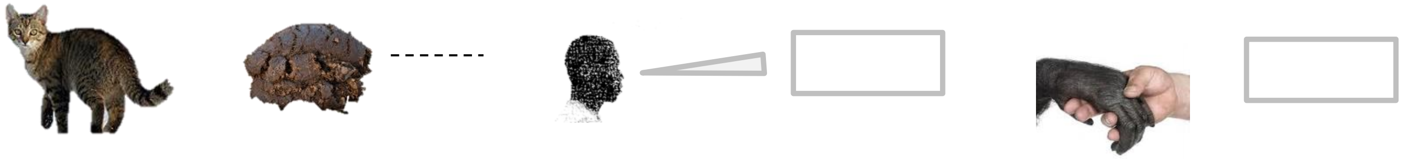

d) Dog

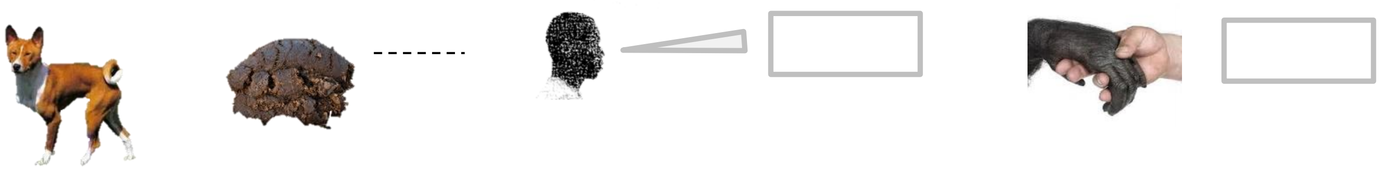

File number ________ | Day ___ of 7

45. Did you see gorillas today?

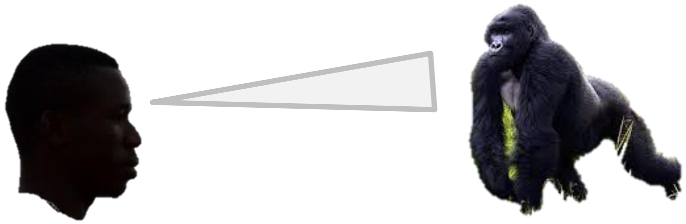

✓

a) Yes ....
b) No ....
c) Prefer not to answer ..

46. Did a gorilla touch you?

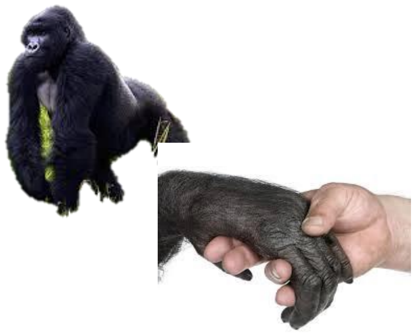

✓

a) Yes ....
b) No ....
c) Prefer not to answer ..

File number ________ | Day ___ of 7

47. Did you see around your house or touch:

a) Monkey

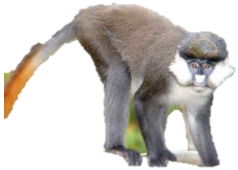
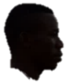 ✓ ☐
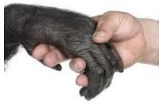 ✓ ☐

b) Bushpig

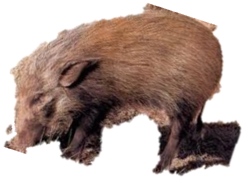
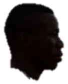 ☐
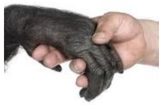 ☐

c) Baboon

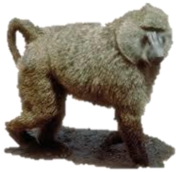
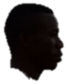 ☐
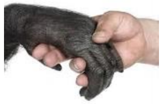 ☐

d) Civet

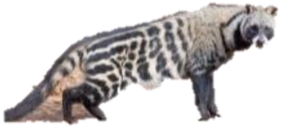
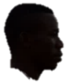 ☐
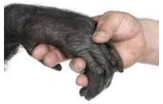 ☐

e) Chimpanzee

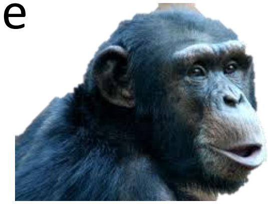
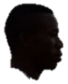 ☐
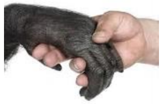 ☐

File number ________ | Day ___ of 7

48. Did you see around your house or touch:

				✓		✓
a) Elephant	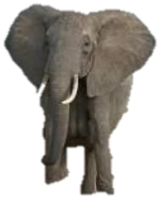	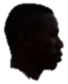	☐	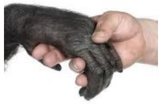	☐
b) Colobus	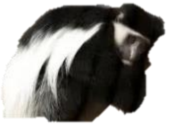	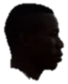	☐	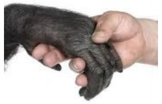	☐
c) Porcupine	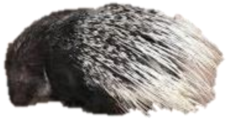	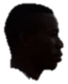	☐	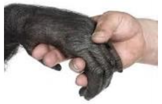	☐
d) Duiker	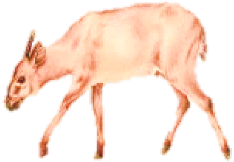	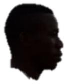	☐	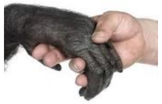	☐
e) Bushbuck	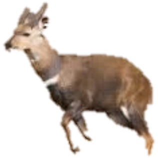	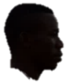	☐	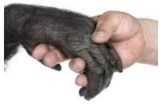	☐
f) Squirrel	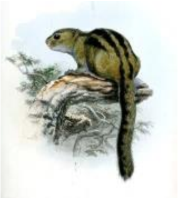	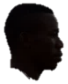	☐	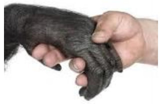	☐

49. Today have you seen the following touch or got very close (e.g. be together in the same field, trees or plantation):

File number ________ |

Day ___ of 7

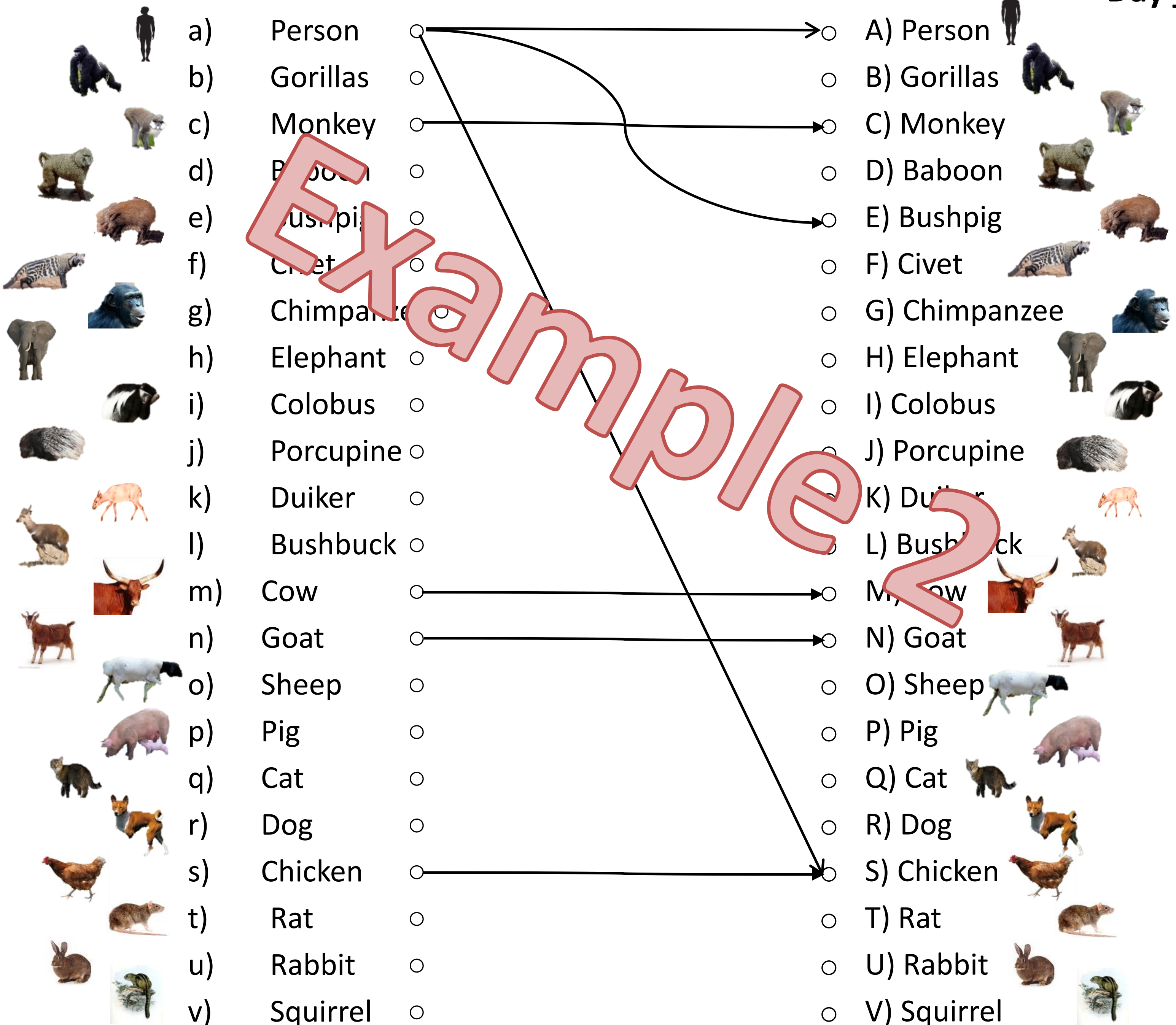

File number ________ |

Day ___ of 7

49. Today have you seen the following touch or got very close (e.g. be together in the same field, trees or plantation):

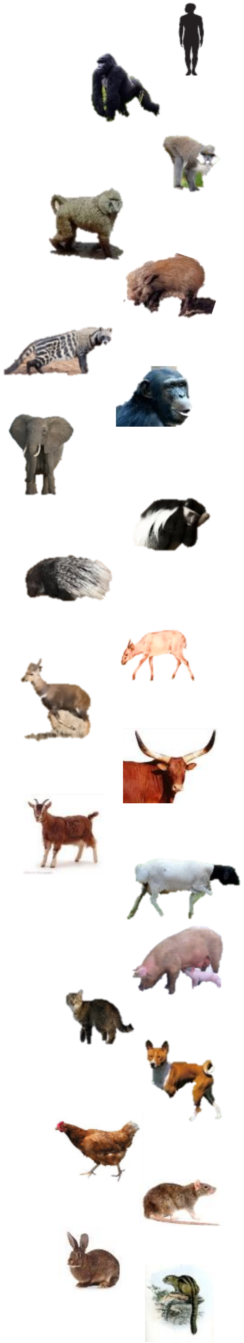

a) Person	○	○	A) Person
b) Gorillas	○	○	B) Gorillas
c) Monkey	○	○	C) Monkey
d) Baboon	○	○	D) Baboon
e) Bushpig	○	○	E) Bushpig
f) Civet	○	○	F) Civet
g) Chimpanzee	○	○	G) Chimpanzee
h) Elephant	○	○	H) Elephant
i) Colobus	○	○	I) Colobus
j) Porcupine	○	○	J) Porcupine
k) Duiker	○	○	K) Duiker
l) Bushbuck	○	○	L) Bushbuck
m) Cow	○	○	M) Cow
n) Goat	○	○	N) Goat
o) Sheep	○	○	O) Sheep
p) Pig	○	○	P) Pig
q) Cat	○	○	Q) Cat
r) Dog	○	○	R) Dog
s) Chicken	○	○	S) Chicken
t) Rat	○	○	T) Rat
u) Rabbit	○	○	U) Rabbit
v) Squirrel	○	○	V) Squirrel

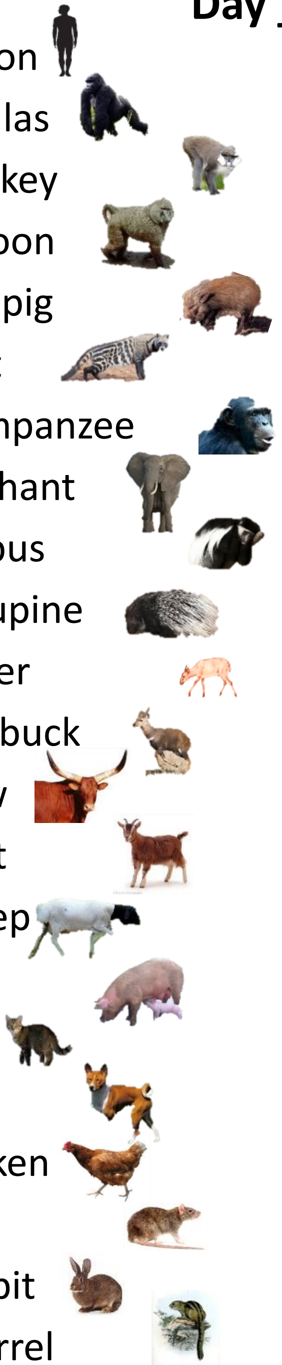

Supplement: S2 File — (PDF) [file pone.0254467.s014.pdf]
